# Bioinformatics analysis of the proteins interacting with LASP-1 and their association with HBV-related hepatocellular carcinoma

**DOI:** 10.1038/srep44017

**Published:** 2017-03-07

**Authors:** Fan-Yun Kong, Ting Zhu, Nan Li, Yun-Fei Cai, Kai Zhou, Xiao Wei, Yan-Bo Kou, Hong-Juan You, Kui-Yang Zheng, Ren-Xian Tang

**Affiliations:** 1 Jiangsu Key Laboratory of Brain Disease Bioinformation, Xuzhou Medical University, Xuzhou, Jiangsu, China; 2 Department of Pathogenic Biology and Immunology, Jiangsu Key Laboratory of Immunity and Metabolism, Xuzhou Medical University, Xuzhou, Jiangsu, China.

## Abstract

LIM and SH3 domain protein (LASP-1) is responsible for the development of several types of human cancers via the interaction with other proteins; however, the precise biological functions of proteins interacting with LASP-1 are not fully clarified. Although the role of LASP-1 in hepatocarcinogenesis has been reported, the implication of LASP-1 interactors in HBV-related hepatocellular carcinoma (HCC) is not clearly evaluated. We obtained information regarding LASP-1 interactors from public databases and published studies. Via bioinformatics analysis, we found that LASP-1 interactors were related to distinct molecular functions and associated with various biological processes. Through an integrated network analysis of the interaction and pathways of LASP-1 interactors, cross-talk between different proteins and associated pathways was found. In addition, LASP-1 and several its interactors are significantly altered in HBV-related HCC through microarray analysis and could form a complex co-expression network. In the disease, LASP-1 and its interactors were further predicted to be regulated by a complex interaction network composed of different transcription factors. Besides, numerous LASP-1 interactors were associated with various clinical factors and related to the survival and recurrence of HBV-related HCC. Taken together, these results could help enrich our understanding of LASP-1 interactors and their relationships with HBV-related HCC.

LIM and SH3 domain protein (LASP-1) is a scaffold protein that has been identified to facilitate the development of several types of human cancers^1^, including breast carcinoma^2^, prostate carcinoma^3^, colorectal carcinoma^4^, gastric cancer^5^, oesophageal squamous cell carcinoma^6^, and gallbladder cancer^7^. Functional experiments of LASP-1 indicate that it plays critical roles in cell migration, invasion, proliferation, epithelial-mesenchymal transition (EMT), cell cycle and signalling pathways^1^,^4^,^5^,^8^,^9^,^10^. Moreover, current clinical studies suggest that over-expression of LASP-1 could serve as a prognostic marker and is correlated with increased clinical stage, lymph node metastasis, and poor survival of cancer patients^1^. Given the importance of LASP-1 function and its clinical relevance in different cancers, LASP-1 might be used as a potential molecular target for the clinical treatment of patients with different tumours.

Structurally, LASP-1 contains an N-terminal LIM domain, two nebulin-like repeats named R1 and R2 domain, and a C-terminal SH3 domain^1^,^11^. These unique domains facilitate its interaction with a variety of proteins. To date, several binding partners of LASP-1, such as VASP, zyxin, Krp1, and CXCR2^1^, have been reported. However, the precise molecular functions and physiological processes associated with LASP-1 interactors have been not fully clarified. Recently, Shao Z. *et al*. reported that LASP-1 interacts with 14–3–3σ in colorectal cancer (CRC) cells and contributes to the progression and metastasis of CRC via the inhibition of 14-3-3σ expression^12^. In addition, a study from the same group showed that LASP-1 not only interacts with S100 calcium binding protein A11 (S100A11) but also increases its expression in CRC cells. Furthermore, the interaction of LASP-1 with S100A11 is required for EMT as well as progression of CRC^13^. Taken together, these results indicate that elucidating the interaction of LASP-1 with its binding partners will help us to further understand the molecular mechanism of LASP-1 on the development of different types of cancer.

LASP-1 over-expression is also observed in hepatocellular carcinoma (HCC) and associated with poor clinical prognosis of the disease^14^. In addition, different cellular factors, such as P53 and uPA, participate in the regulation of LASP-1 expression in HCC cells^15^,^16^. In addition, the results from Wang H *et al*. indicate that the increase of LASP-1 in HCC tissues is related to hepatitis B virus (HBV) infection^14^. Furthermore, we previously discovered that HBV X protein (HBX) was responsible for the upregulation of LASP-1 in HCC cells^17^. Currently, Salvi A. *et al*. identified that vimentin is a new binding partner of LASP-1 in HCC cells^18^. However, the binding partners involved in the development of HBV-related hepatocellular carcinoma mediated by LASP-1 are not completely understood. In this study, we obtained data of LASP-1 interacting proteins from public databases and published studies and assessed their biological functions, associated pathways, and interaction networks via bioinformatics analysis. Furthermore, the gene expression and potential regulatory factors of LASP-1 interactors were further investigated using a microarray of HBV-related HCC tissues that was downloaded from the NCBI gene expression omnibus (GEO) database to identify candidate genes that may contribute to the development of HBV-related HCC in conjunction with LASP-1.

## Results

### Information regarding LASP-1 interacting proteins

To investigate the proteins that interact with LASP-1, the information of target human proteins was retrieved from different public databases, including IntAct^19^, BioGRID^20^, APID^21^, PINA2.0^22^, Mentha^23^, HitPredict^24^, WiKi-Pi^25^, PIPs^26^, PPI-finder^27^ and PrePPI^28^ or from the studies reported in PubMed. A total of 390 predicted or experimentally validated LASP-1 interacting proteins were obtained. Given that predicated LASP-1 interactions in many databases are mainly based on indirect clues, such as data mining in the PPI-finder database^27^, Bayesian prediction in the PIPs database^26^, and a prediction method mainly based on structural information of target proteins in the PrePPI database^28^, it is difficult to determine the accuracy or reliability of predicted results from these indirect evidences. Furthermore, as mentioned by Zhang QC. *et al*.^28^, many of the interactions that form computational predictions are often more indicative of functional associations between two proteins than of direct physical interactions. Additionally, most of the current studies related to computational analysis of protein interactions or the interactome are mainly dependent on experimentally derived proteins^29^,^30^,^31^,^32^. Therefore, the proteins that interact with LASP-1 based on computational predictions but without experimental validation (Supplementary Table S1) were removed. As shown in Table 1, a total of 89 LASP-1 interacting proteins were ultimately included in the study.

Among these proteins, 79 proteins were obtained from different public databases, and these proteins were mainly identified by different methods, such as co-fractionation, affinity capture-MS, proximity label-MS, and two-hybrid array. Ten proteins, including CXCR1^33^, KLHL41^34^, PALLD^35^, VASP^36^, VIM^18^, S100A11^13^, and SFN^12^, were mainly extracted from the studies reported in PubMed. The interaction of the 10 proteins with LASP-1 reported in different studies was mainly certified with co-immunoprecipitation. In addition, most of the gene and protein symbols were identified in the UniProtKB database^37^, except the protein B3KY43, which was obtained from HitPredict database. The information on B3KY43 was found in the UniProtKB database, but no definite gene symbol and UniProt accession number was available.

### Protein class and location of LASP-1 interacting proteins

**Figure 1:**
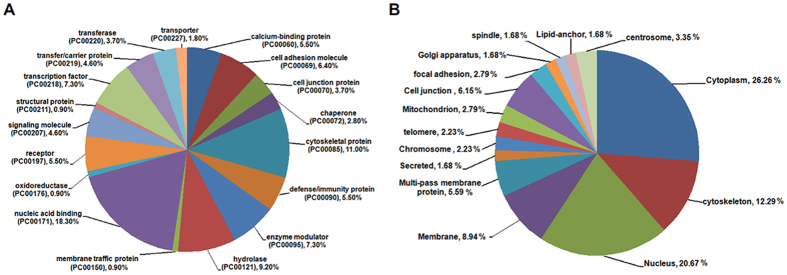
The protein class and location of LASP-1 interactors. **(A)** The protein class of LASP-1 interacting proteins identified by the PANTHER classification system. **(B)** The cellular location of LASP-1 interactors investigated using the UniProtKB database.

Next, we assessed the protein class of LASP-1 interactors using the PANTHER classification system^38^. As shown in Fig. 1A and Supplementary Table S2, LASP-1 interactors are associated with different protein types, including nucleic acid binding, cytoskeletal protein, hydrolase, transcript factor, defence/immunity protein, calcium-binding protein, and receptors. In addition, the cellular localization information of LASP-1 interactors was investigated using the UniProtKB database^37^. Proteins that interact with LASP-1 are mainly situated in the cellular cytoplasm, nucleus, cytoskeleton and membrane. However, a few of LASP-1 interactors are also located in the special areas, such as the telomere, Golgi apparatus and lipid anchor (Fig. 1B and Supplementary Table S3).

### The molecular function and pathway enrichment analysis of LASP-1 interactors

**Figure 2:**
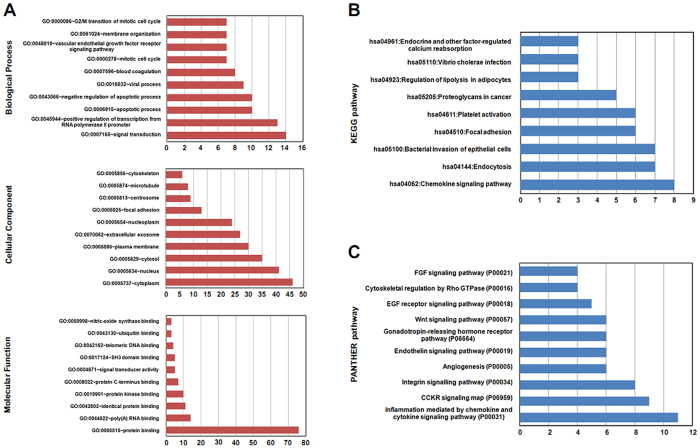
The top 10 enrichment function and pathway terms of LASP-1 interactors. **(A)** The top 10 GO terms of LASP-1 interacting proteins included biological process, cellular component, and molecular function. **(B)** The pathway analysis of LASP-1 interactors identified by the KEGG classification system. **(C)** The top 10 pathways associated with protein interacted with LASP-1 as assessed by PANTHER pathway analysis.

In the study, gene function annotation of LASP-1 interactors was performed with GO analysis using the Database for Annotation, Visualization and Integrated Discovery (DAVID, v6.8 tool)^39^,^40^. The results suggest that LASP-1 interactors are mostly enriched in several biological processes, cellular components, and molecular functions (Supplementary Table S4). As shown in Fig. 2A, LASP-1 interactors mainly participated in signal transduction, the positive regulation of transcription from the RNA polymerase II promoter, apoptotic processes, and the negative regulation of apoptotic processes. In addition, GO analysis demonstrates that LASP-1 interactors are located in different areas within the cell, such as cytoplasm, nucleus, cytosol, and membrane. In addition, GO annotation suggests that LASP-1 interactors are mainly enriched in molecular functions of proteins binding with protein, RNA and DNA.

The pathways that LASP-1 interactors were involved in were assessed by KEGG pathway and PANTHER pathway. The two different pathway enrichment systems identified many different pathways associated with LASP-1 interactors (Supplementary Tables S5 and 6). For example, the results of KEGG pathways show that LASP-1 interactors are mainly involved in the chemokine signalling pathway, endocytosis, and bacterial invasion of epithelial cells (Fig. 2B). The PANTHER pathway’s results suggest that LASP-1 interacting proteins are mainly associated with inflammation mediated by chemokine and cytokine signalling pathways, CCKR signalling map, and the integrin signalling pathway (Fig. 2C).

### Network integration of the interaction and pathways associated with LASP-1 interactors

**Figure 3:**
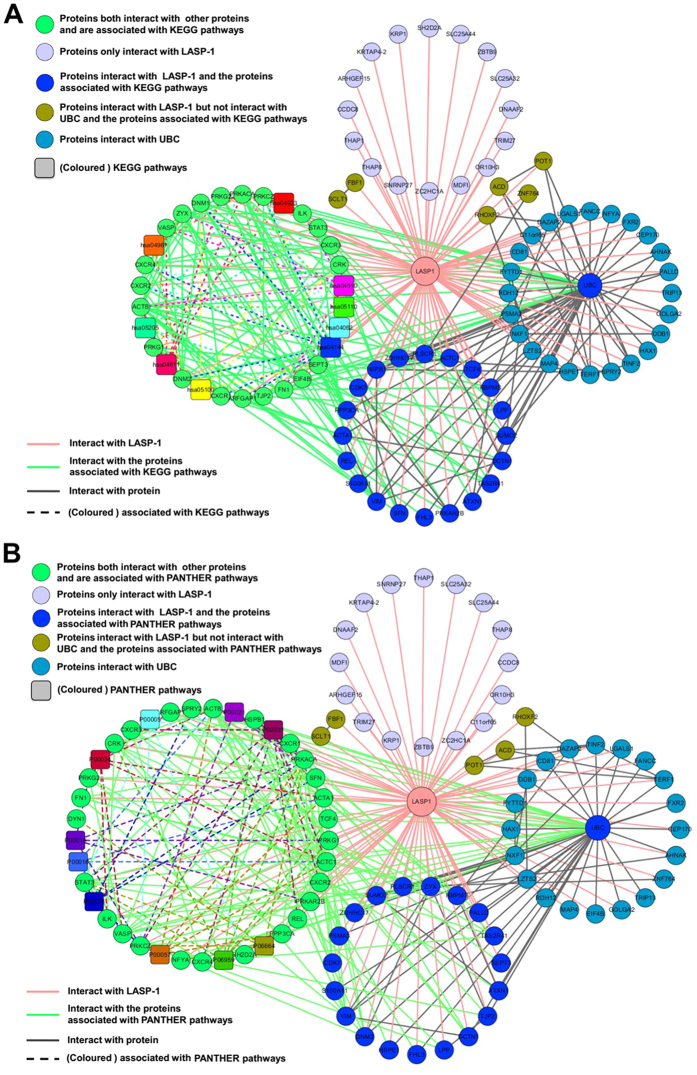
The integration network of interaction and pathways of LASP-1 and its interactors visualized by Cytoscape 3.2.1 software. (**A**) The integration of interaction and KEGG pathways of LASP-1 and its interactors. (**B**) The integration of interaction and the top 10 PANTHER pathways of LASP-1 and its interactors.

To better understand the interaction of LASP-1 with its interactors and the crosstalk of these proteins with annotated pathways, we constructed the interaction network containing nodes corresponding to LASP-1 with its interacting proteins and further integrated the interaction information of LASP-1 interactors with their associated pathways. The interaction data of LASP-1 and its interactors was not only extracted from STRING tools^41^ (Supplementary Table S7) but also extracted from the data collected in this study (Table 1). As shown in Fig. 3, using the graph-visualization tool Cytoscape 3.2.1^42^, we found that LASP-1 and its interactors could form complex networks. In addition, the protein Ubiquitin C (UBC) is significant hub protein that could interact with many additional proteins in the interaction network. Among these LASP-1 interacting proteins, 21 proteins were annotated with 9 different KEGG pathways (Fig. 3A), and 27 proteins were involved in the top 10 PANTHER pathways (Fig. 3B). In addition, LASP-1 and a lot of its interacting proteins could cross-talk with these KEGG pathways or PANTHER pathways via the interaction with proteins that are involved in these pathways, and these annotated pathways were also closely interconnected by “cross-talk” proteins. Taken together, these results imply that LASP-1 and its interacting proteins perform various functions via the interaction with the proteins that are involved in various pathways.

### The expression of LASP-1 interactors and predicted transcription factors in HBV-related HCC

**Figure 4:**
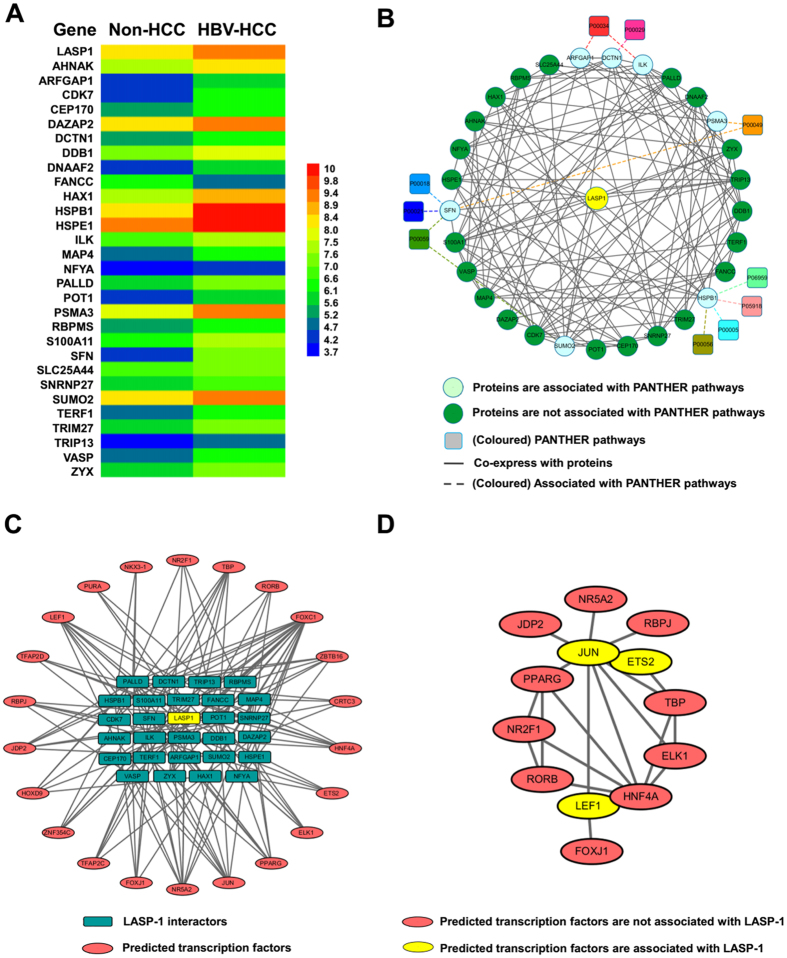
The relative expression and predicted transcription factors of LASP-1 interactors in HBV-related HCC tissues. **(A)** The relative expression of LASP-1 interactors with a fold-change of approximately 2 and P < 0.05. **(B)** The integration networks of co-expression and PANTHER pathways of LASP-1 and its interactors visualized by Cytoscape 3.2.1 software. **(C)** The predicted transcription factors of LASP-1 and its interacting proteins. **(D)** The interactome networks of predicted transcription factors.

Since the over-expression of LASP-1 has been reported in HCC^15^,^16^,^18^ and is associated with HBV infection in HCC patients^14^, we investigated whether the LASP-1 interactors are also associated with HBV-related HCC. Based on the gene expression profiling analysis of GSE14520^43^,^44^,^45^ described before, we found that the change in LASP-1 was greater than 2.0-fold with a P-valve < 0.05 in HBV-HCC tissues compared with non-HCC tissues (Fig. 4A). We next assessed the expression of all genes of LASP-1 interactors in HBV-related HCC tissues (Supplementary Table S8). The information from genes of LASP-1 interactors with similar changes of LASP-1 (2.0-fold change, P-value < 0.05) in HBV-related HCC tissues were extracted for further analysis. For the multiple probes mapped to the same gene in the microarray, if at least one probe value of the gene was altered greater than 2.0-fold with a P-value less than 0.05, the gene was also enrolled in present study. Then, the median was calculated based on values of multiple probes corresponding to the same gene. As shown in Fig. 4A, compared with non-HCC tissues, the expression of a total of 29 genes of LASP-1 interactors was significantly altered in HBV-HCC tissues. Among these genes, only FANCC was downregulated in HBV-related HCC, and the expression levels of other 28 genes were increased in the diseased cells (Fig. 4A and Supplementary Table S8). We further investigated the co-expression status of LASP-1 interactor genes in HBV-related HCC tissues. In addition, Pearson’s correlation coefficients (≥0.80) were applied to identify the co-expression relationship of LASP-1 interactors, and 127 co-expression pairs of LASP-1 with its interactors were identified (Supplementary Table S9). Next, the networks of the co-expression of LASP-1 interactors with their associated pathways were integrated through Cytoscape 3.2.1 software. Our data showed that LASP-1 and all of the 29 LASP-1 interactors could form a complex co-expression network. In addition, through pathway analysis, we found that these 29 proteins were not annotated with significantly enriched KEGG pathways, whereas 7 proteins were associated with 10 PANTHER pathways, implying that the abnormal alteration of these 10 pathways were involved in the development of HBV-related HCC (Fig. 4B).

Given that the co-expression network of LASP-1 and its interactors was present in HBV-related HCC tissues, we explored whether the expression of LASP-1 interactors was regulated by the interaction of distinct transcription elements. Via transcription factor enrichment analysis using the Enrichr tool^46^,^47^, we found that LASP-1 and its interactors could form a complex network with a total of 22 transcription factors (Fig. 4C and Supplementary Table S10). Within the network, one transcription factor target more than three LASP-1 interactor genes, and one LASP-1 interactor was mainly controlled by at least three transcription factors. Because current studies show that the function of transcription factors is based on the interaction with other proteins^48^, we investigated whether the expression of LASP-1 and its interactors associated with HBV-related HCC was potentially regulated by the interaction of different transcription factors. Using the STRING platform, we found that 13 transcription factors interact with others (Supplementary Table S11) and form a complex interaction network (Fig. 4D). Taken together, these results implied that LASP-1 and its interactors could be potentially regulated by a complex interaction network composed of different transcription factors in HBV-related HCC. Furthermore, in this network, ETS2, LEF1 and JUN were the predicted transcription factors that are directly responsible for LASP-1 expression. We also measured the relative expression of predicted transcription factors in a microarray of HBV-related HCC tissues. With the exception of ETS2, JUN and ZBTB16, the expression of most of these transcription factors was upregulated (average changes greater than 1.2-fold) in HBV-related HCC, compared with non-HCC (Supplementary Table S12). These results suggest that the aberrant alteration of transcription factors was associated with the collaborative changes of LASP-1 with its interactors in HBV-related HCC.

### The association of LASP-1 interactors with clinical factors of HBV-related HCC patients

**Figure 5:**
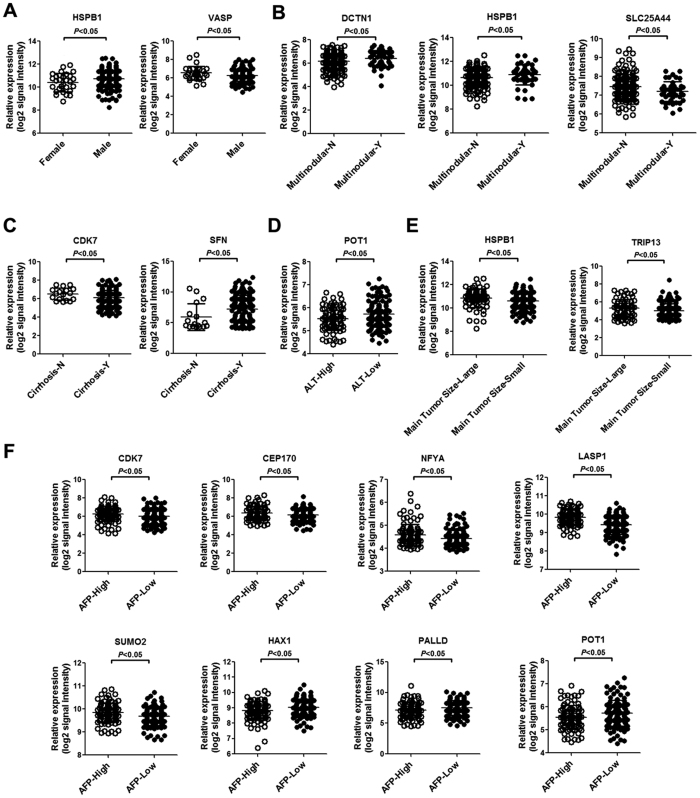
The expression of LASP-1 and its interactors in HBV-related HCC patients with various clinical factors. **(A)** The expression of HSPB1 and VASP in male and female HBV-related HCC patients. **(B)** The expression of DCTN1, HSPB1 and SLC25A44 in patients with multinodular or without multinodular HCC. **(C)** The expression of CDK7 and SFN in patients with or without cirrhosis. **(D)** The expression of POT1 in HBV-related HCC patients with high and low ALT levels. **(E)** The expression of HSPB1 and TRIP13 in patients with large or small main tumour sizes. **(F)** The expression of CDK7, CEP170, NFYA, LASP-1, SUMO2, HAX1, PALLD and POT1 in patients with high or low AFP levels. ALT-High represents patients with high ALT levels (>50 U/L) and ALT-Low represents patients with low ALT levels (≤50 U/L). Cirrhosis-N represents patients without cirrhosis, and Cirrhosis-Y represents patients with cirrhosis. Main Tumour Size-Large indicates patients with a large main tumour size (>5 cm), Main Tumour Size-Small indicates patients with a small main tumour size (≤5 cm). AFP-High represents patients with high AFP levels (>300 ng/ml), AFP-Low represents patients with low AFP levels (≤300 ng/ml).

We investigated the association of LASP-1 and its interactors with clinical parameters, including gender, HBV viral status, ALT, main tumour size, multinodular disease, cirrhosis, BCLC staging and AFP, in HBV-related HCC tissues. As shown in Fig. 5A, compared with female HBV-related HCC patients, the expression of HSPB1 was significantly increased in male patients, whereas the expression of VASP was reduced in male patients. Compared with patients without multinodular disease, the expression levels of DCTN1 and HSPB1 were increased in patients with multinodular disease. However, in patients with multinodular disease, the expression of SLC25A44 was reduced compared to patients without multinodular disease (Fig. 5B). Compared with patients without cirrhosis, the expression of CDK7 was downregulated in patients with cirrhosis, whereas the expression of SFN was upregulated in patients with cirrhosis (Fig. 5C). Compared with patients with high ALT levels, POT1 expression was significantly increased in patients with low ALT levels (Fig. 5D). In patients with a large main tumour size, the expression of HSPB1 and TRIP13 was increased compared to patients with a small main tumour size (Fig. 5E). In addition, we observed that the expression of CDK7, CEP170, NFYA, LASP-1 and SUMO2 was significantly increased in patients with high AFP levels compared with patients with low AFP levels. However, compared to patients with low AFP, the expression levels of HAX1, PALLD and POT1 were reduced in patients with high AFP levels (Fig. 5F).

### The relationship of LASP-1 interactors with overall survival and recurrence of HBV-related HCC patients

Using the Cox regression model in the multivariate analysis via SPSS 16.0 software, we further investigated the association of LASP-1 interactors with clinical outcomes, such as survival and recurrence, in patients with HBV-related HCC. The results show that among these LASP-1 interactors, SLC25A44 and SNRNP27 are not only related to poor survival but also associated with disease recurrence (Tables 2 and 3). In addition, ILK, NFYA and RBPMS are related to poor survival of HBV-related HCC (Table 2).

## Discussion

The function of LASP-1 is based on the interaction with other proteins. However, the precise molecular functions and physiological processes involved in proteins interacting with LASP-1 are not clear. Current studies suggest that LASP-1 is involved in the progression of HCC with HBV infection^14^; however, whether LASP-1 interacting proteins contribute to the development of HBV-related HCC is largely unknown. In the present study, we obtained information regarding proteins that interacted with LASP-1 from public databases and published studies. Based on bioinformatics analysis, the LASP-1 interactors were found to exert distinct molecular functions that are involved in a variety of biological processes and could form a complex interaction network. In addition, LASP-1 and several of its interactors were overexpressed in a complex co-expression pattern in HBV-related HCC. Furthermore, LASP-1 and its interactors that are altered in HBV-related HCC were predicted to be regulated by a complex interaction networks composed of different transcription factors. Furthermore, some LASP-1 interactors are associated with various clinical factors and related to the survival and recurrence of HBV-related HCC.

Currently, the implication of LASP-1 in several types of cancers has been reported^1^. Although LASP-1 is found to mediate cell proliferation, migration, and EMT and is associated with poor progression of different types of tumours, the precise molecular functions of LASP-1 are not fully elucidated. LASP-1 contains LIM, R1, R2 and SH3 domains, and these domains are capable of interacting with a variety of proteins^1^,^11^. In addition, the function of LASP-1 is dependent on the interaction with its binding partners. Therefore, clarifying the characteristics of LASP-1 interactors helps us further understand the biological roles of LASP-1. In this study, we obtained the information of LASP-1 interacting proteins from online databases or published studies in PubMed. In databases IntAct^19^, BioGRID^20^, APID^21^, PINA2.0^22^, Mentha^23^, HitPredict^24^ and WiKi-Pi^25^, the protein interaction data were mainly based on experimentally evidences that derived from published literatures and direct user submissions, or extracted from other databases. One limitation of these databases is that the backend databases are needed to be updated whenever there are new data released from literatures or other databases. The data from databases PIPs^26^, PPI-finder^27^ and PrePPI^28^ are relied on computational methods, including Bayesian method that combines information of protein expression, post-translational modifications, domain co-occurrence and sub-cellular location^26^; text-mining based on their co-occurrences and interaction words^27^; and the approach based on calculated likelihood ratios that combining the protein information of evolutionary, structural, functional, and expression^28^. Because each computational method is under different standard, the prediction coverage and accuracy of protein-protein interaction in various databases are different^49^,^50^, which lead to the extremely heterogeneous of data. Experimentally data is considered to be of higher quality than data obtained by computational methods, although errors and false positives in data capture from experimentally methods may be still occur^51^. In order to ensure the reliability of data and the accuracy of bioinformatics analysis, the proteins from computational prediction but without experimental validation in our used databases were excluded.

Previous reports show that LASP-1 mainly interacts with cytoskeletal-associated proteins, such as F-actin, Krp1, LPP, VASP and zyxin, and is located in cytoplasm, nucleus, membrane and multiple sites of dynamic actin assembly, such as focal contacts, focal adhesions and pseudopodia^1^,^11^. However, LASP-1 exhibits cell-specific inhomogeneous expression patterns in different types of tumours. For example, LASP-1 is present in focal contacts, and the cytosol, as well as perinuclear and nuclear breast cancer cells^52^. Our previous study showed that LASP-1 is mainly located in pseudopods and the cytoplasm of HCC cells^17^. Given that LASP-1 interactors, such as PALLD, play an important role on proper subcellular localization of LASP-1^35^, it is reasonable to hypothesize that the subcellular localization of LASP-1 is mainly dependent on the expression pattern of its interactors in different cells and the expression pattern of LASP-1 interactors is cell type-specific. Based on the analysis of protein class and protein location through PANTHER and UniProtKB databases, the protein types that interact with LASP-1 were identified, and a number of identified proteins were situated in the cytoplasm, nucleus and membrane, which are similar to the areas of LASP-1 localization. In addition, we found that a small amount of identified proteins are located in various special areas, including the telomere, Golgi apparatus and lipid anchor, implying that these proteins might recruit LASP-1 to special areas and exert important biological functions in particular types of cells.

We next investigated the enriched distinct molecular functions and biological pathways through GO, KEGG and PANTHER databases. In the present study, multiple GO terms and KEGG and PANTHER pathways were identified for LASP-1 interactors, suggesting that LASP-1 was involved in a variety of molecular functions and pathways by interacting with its binding partners. In GO biological processes, LASP-1 interactors are associated with signal transduction and regulation of apoptosis, and these biological processes are consistent with previous reports that LASP-1 plays key roles in apoptosis regulation and cell signalling^1^. Regarding the cellular component of the GO term, different cellular areas are related to LASP-1 interactors, and most of cellular components are consistent with the results of the protein class analysed by the PANTHER database. Although a variety of molecular functions were associated with LASP-1 interactors, these molecular functions are mainly associated with protein, RNA, and DNA binding, suggesting that most of the LASP-1 interactors are scaffold proteins involved in protein-protein interaction, protein-DNA interaction, and protein-RNA interaction. Although several biological processes, cellular components, and molecular functions in the GO terms of LASP-1 interactors shown in Fig. 2A have not been reported previously, these distinct enriched GO terms may help us to further explore the biological functions of LASP-1 and its binding partners in different cell types.

In addition, via KEGG and PANTHER pathway analysis, various pathways are identified to be associated with LASP-1 interacting proteins, and the distinct enriched KEGG pathways of LASP-1 interactors suggests that these proteins are mainly involved in the chemokine signalling pathway, endocytosis, and bacterial invasion of epithelial cells, while the results of the PANTHER pathway analysis indicate that LASP-1 interacting proteins are associated with inflammation mediated by the chemokine and cytokine signalling pathways, CCKR signalling map, and integrin signalling pathway. It is a well-known fact that the pathway information, annotation, nomenclature, and update vary between KEGG and PANTHER pathway databases. In addition, as mentioned by Chowdhury S *et al*.^53^, the technical details associated with computational tools for users to search and analyse widely differ between these two databases. Therefore, it is not surprising to observe the heterogeneity of pathway information from KEGG and PANTHER pathway databases using the proteins interacting with LASP-1. Given that LASP-1 and its interactors were associated with a variety of pathways, the abnormal expression of LASP-1 and its interactors in disease states might alter the associated pathways. Regarding the diseases associated with LASP-1 and its interactors, targeting the specific pathways identified in this study may be a potential strategy.

We further constructed an integrated network containing both the interaction of LASP-1 with its interacting proteins and the association of LASP-1 interactors with their annotated pathways. Based on the integrated network analysis, Ubiquitin C was found to be a significant hub protein that could interact with LASP-1 and many of its interactors. Since Ubiquitin C plays a critical role in protein degradation through ubiquitination pathway to modulate the activity and function of targeted proteins^54^, the results in our study imply that ubiquitination pathway is an important mechanism for regulating the biological functions of LASP-1 and its interactors. Furthermore, current studies show that Ubiquitin C is involved in the development and drug resistance of cancer with different types^55^,^56^,^57^. Therefore, further exploring the functional regulation of LASP-1 and its interacting proteins by Ubiquitin C-associated ubiquitination pathway in different tumours will help us better understand the molecular mechanism of carcinogenesis associated with LASP-1 and its interactors.

Recently, the aberrant over-expression of LASP-1 has been reported in HCC tissues^15^,^16^,^18^. In particular, LASP-1 is associated with HBV infection in HCC patients^14^. However, the expression of LASP-1 interactors in association with HBV-related HCC is not well clear. By analysing the gene expression profile of HBV-related HCC and non-HCC tissues in GSE14520 downloaded from GEO database^43^,^44^, the expression of a total of 29 LASP-1 interactors were altered with similar fold-changes as LASP-1. In addition, the results of the co-expression analysis indicated that these genes could form a complex co-expression network in HBV-related HCC. Within these LASP-1 interactors, S100A11^58^, zyxin^59^, HAX-1^60^, and ILK^61^ are over-expressed in HCC and contribute to HCC cell invasion or proliferation. In addition, HBX is capable of inducing the upregulation of S100A11 and PSMA3^62^. In addition, the study from Li H *et al*. indicates that ARFGAP1 is a new factor for HCV replication via the interaction with HCV NS5A in HCC cells^63^. These results indicate that LASP-1 interactors are not associated with hepatocarcinogenesis, but their activities could be mediated by hepatitis virus proteins. Further exploring the roles and associated regulatory mechanisms of LASP-1 interactors in HBV-related HCC may help us to identify candidate anti-cancer targets for treatment of the disease.

Gene expression is mediated by transcription factors, and several transcription factors are functionally altered in HCC and participate in the development of liver cancer. In this study, we assessed whether LASP-1 and its interactors were controlled by distinct transcription factors in HBV-related HCC. The results suggested that the expression of target genes was mainly controlled by 22 predicted transcription factors. Current studies suggest that cooperativity between transcription factors is vital in the regulation of gene expression^48^. In addition, with the development of high-throughput technology and computer prediction methods, several interactions with transcription factors were identified^64^,^65^,^66^. In present study, we explored whether these transcription factors could form potential gene expression regulatory networks. As expected, based on the protein network tool STRING, we found that these transcription factors could also interact with each other, implying that the expression of LASP-1 and it interactors was potentially controlled by a complex interaction network composed of 13 transcription factors.

We also measured the expression levels of the 22 predicted transcription factors in HBV-related HCC tissues via microarray data from GSE14520. Compared with non-HCC, an at least 1.2-fold change was noted for most transcription factors in HBV-related HCC. Previous reports indicate that a value of 1.2 is considered a low and acceptable cut-off for fold-change with statistical significance in microarrays^67^, suggesting that these predicted transcription factors are also significantly dysregulated in HBV-related HCC. As expected, among these predicted transcription factors, the over-expression of FOXJ1^68^, FOXC1^69^ and HOXD9^70^ has been observed in HCC tissues by different research groups. In particular, HBX was responsible for the upregulation of PPARG gene^71^. In addition, Tian X *et al*. reported that LEF1 is increased in HBV-associated HCCs^72^, and Hong M *et al*. found that HNF-4α facilitated HBV replication in HCC cells^73^. Taken together, these results suggest that the predicted transcription factors play an important role in HBV-related HCC through mediating the over-expression of LASP-1 and its interactors.

The association of LASP-1 and its interactors with clinical factors, survival and the recurrence of HBV-related HCC were further investigated. Our results indicate that many LASP-1 interactors are associated with different clinical factors, including gender, multinodular, cirrhosis, ALT, main tumour size and AFP, implying that LASP-1 interactors might play critical roles in the abnormalities of these clinical factors in HBV-related HCC patients. Furthermore, the results of multivariate analysis with the Cox regression model show that SLC25A44 and SNRNP27 are not only associated with overall survival of HBV-related HCC but also related to recurrence of the disease. In addition, ILK, NFYA and RBPMS are associated with poor survival of HBV-related HCC. These results suggest that LASP-1 interactors could be used as biomarkers of poor clinical prognosis of HBV-related HCC patients. Current research from Wang H *et al*. indicates that LASP-1 is a potential prognostic factor of HCC^14^. Integration of the expression information of LASP-1 with its interactors with suitable methods may be a better strategy for HBV-related HCC monitoring and management to reduce mortality and prolong the survival time.

In conclusion, based on bioinformatics analysis, we investigated the characteristics, associated molecular functions and pathways of LASP-1 interactors obtained from public databases or published studies, and a variety of different LASP-1 interactors were identified. In addition, LASP-1 interacting proteins are not only associated with distinct functions and pathways but could also form complex networks. In addition, LASP-1 and its interactors are altered in HBV-related HCC tissues and potentially controlled by a complex regulatory network composed of different predicted transcription factors. Furthermore, various LASP-1 interactors are significantly associated with clinical factors and related to the poor progress of HBV-related HCC. Given the distinct functions and clinical significance of LASP-1 and its interactors, both LASP-1 and its interactors could be used as novel biomarkers or therapeutic targets for patients with HBV-related HCC.

## Materials and Methods

### Collection of LASP-1 interactors

The proteins, which have been predicted or experimentally detected to interact with LASP-1, were extracted from the public databases, including IntAct^19^, BioGRID^20^, APID^21^, PINA2.0^22^, Mentha^23^, HitPredict^24^, WiKi-Pi^25^, PIPs^26^, PPI-finder^27^ and PrePPI^28^, or retrieved from published literatures in PubMed. Non-human interactors of LASP-1 extracted from these databases were removed from further analysis. The gene symbols and protein symbols were identified using the UniProtKB database^37^.

### Protein class and location analysis

The PANTHER classification system was used to identify the protein class of LASP-1 interactors^38^. In addition, the analysis of protein location of LASP-1 interactors was performed with the UniProtKB database based on the UniProtKB record “Subcellular location”.

### Gene function and pathway enrichment analysis

Gene ontology (GO) analysis was used to identify the enriched molecular functions of clustered genes of LASP-1 interactors. Kyoto Encyclopedia of Genes and Genomes (KEGG) pathway analysis was used to determine the clusters of genes of LASP-1 interactors with associated biological pathways. In addition, the DAVID online tool was applied to perform the enrichment GO and KEGG pathway analysis^39^,^40^. A P-value of <0.05 was considered significant. In addition, we also used the PANTHER classification system to analyse the enriched pathway of cluster genes of LASP-1 interactors.

### Interaction analysis of LASP-1 and its interactors and network integration of LASP-1 interactors with their associated pathways

According to the manufacturer’s instructions, Cytoscape 3.2.1 software^42^ was used to visualize the integration of interaction and pathways of LASP-1 and its interactors. The interaction was analysed based on the information from the following resources: 1) the protein interaction data extracted from STRING^41^,^74^, and 2) the interaction information collected in this study (Table 1). The pathways relied on the information of enriched KEGG pathways and PANTHER pathways. In our maps visualized by Cytoscape 3.2.1 software, the circle nodes represent proteins that could interact with other proteins. Coloured square nodes represent different pathways. Solid edges represent the interactions between different proteins. Coloured dot lines indicate the association with different pathways.

### Microarray source, data processing and identification of differential gene expression

The microarray data GSE14520 was downloaded from the GEO database^43^,^44^. The samples in GSE14520 were mostly HBV-related HCC samples and primarily measured by the Affymetrix HT Human Genome U133A Array. We removed the samples without HBV infection that were not detected with the Affymetrix HT Human Genome U133A Array as previously described^45^. Finally, 212 HBV-HCC and 220 non-HCC cases were analysed in the study, and the patient characteristics and associated factors with clinical outcomes in HBV-related HCC were previously described^45^.

The microarray data were performed with both Affymetrix Expression Console and Affymetrix Transcriptome Analysis Console v3.0 software, according to the manufacturer’s instructions. Using Affymetrix Expression Console and Affymetrix Transcriptome Analysis Console v3.0 software, probe signal values of microarray were converted into log2 values, and genes annotated by the probes were analysed. Data were normalized using the robust multi-array average (RMA) algorithm. The details of data processing and the identification of differential gene expression were previously described^45^.

### Co-expression analysis and network integration of co-expression information of LASP-1 interactors with their associated pathways

The co-expression network analysis of LASP-1 interactors was performed with the DeGNServer online platform in accordance with its operating instructions^75^. For analysing the co-expression status of each pair of genes of LASP-1 interactors, the Pearson correlation was calculated, and paired genes of LASP-1 interactors with a significant correlation based on Pearson correlation coefficient ≥0.80 were selected for further analysis. Cytoscape 3.2.1 software was applied to integrate the co-expression relationship of LASP-1 interactors with their associated pathways. In the maps, the circle nodes represent proteins that could co-express with other proteins. Coloured square nodes represent different pathways. Solid edges represent the co-expressions between different proteins. Coloured dot lines indicate the association with different pathways.

### Transcription factor analysis

The enrichment analysis of transcription factors of LASP-1 interactors was performed using the “TRANSFAC_and_JASPAR_PWMs” section in the Enrichr online tool^46^,^47^. A P-value of <0.05 was considered significant. We used Cytoscape software to visualize the relationship of predicted transcription factors with LASP-1 interactors. In addition, the interaction of one predicted transcription factor with others was further analysed by STRING and visualized with Cytoscape 3.2.1 software.

### Statistical Analysis

Statistical analysis was performed using SPSS 16.0 Software (SPSS Inc., Chicago, USA). Survival analysis was performed with multivariate analysis with the Cox regression model. Data are presented as the mean ± SD and analysed using t tests. P < 0.05 was considered significant.

## Additional Information

**How to cite this article**: Kong, F.-Y. *et al*. Bioinformatics analysis of the proteins interacting with LASP-1 and their association with HBV-related hepatocellular carcinoma. *Sci. Rep.* **7**, 44017; doi: 10.1038/srep44017 (2017).

**Publisher's note:** Springer Nature remains neutral with regard to jurisdictional claims in published maps and institutional affiliations.

## Supplementary Material

Supplementary Information

## Figures and Tables

**Table 1:** The information of LASP1 interactors from online databases and published studies.

Gene symbol	Protein symbol	Uniprot Number	Interaction Detection Method	Sources
ACD	ACD	Q96AP0	Two-hybrid/bimolecular fluorescence complementation	BioGIRD/IntAct/HitPredict/WiKi-Pi/PIAN2/APID/Mentha/PrePPI
ACTA1	ACTS	P68133	*In vitro*	WiKi-Pi/PIAN2/PubMed/PrePPI/PPI-finder
ACTB	ACTB	P60709	*In vivo, in vitro*	WiKi-Pi/PIAN2/APID/PubMed/PrePPI/PPI-finder
ACTC1	ACTC	P68032	*In vivo, in vitro*	WiKi-Pi/PIAN2/APID/PrePPI/PPI-finder
AHNAK	AHNK	Q09666	Co-fractionation	BioGIRD/PIAN2/APID/Mentha/PrePPI
ARFGAP1	ARFG1	Q8N6T3	Co-fractionation	BioGIRD/APID/Mentha/PrePPI
ARHGEF15	ARHGF	O94989	Two-hybrid/two hybrid prey pooling approach	BioGIRD/IntAct/HitPredict/WiKi-Pi/APID/Mentha/PrePPI
ATXN1	ATX1	P54253	Two-hybrid/two hybrid prey pooling approach	BioGIRD/IntAct/HitPredict/WiKi-Pi/APID/Mentha/PrePPI
	B3KY43		*in vitro, in vivo*	HitPredict
C11orf65	CK065	Q8NCR3	Affinity Capture-MS	BioGIRD/APID/Mentha/PrePPI
CCDC8	CCDC8	Q9H0W5	Affinity Capture-MS	BioGIRD/HitPredict/APID/PrePPI
CD81	CD81	P60033	Affinity Capture-MS	BioGIRD/HitPredict/PIAN2/APID/Mentha/PrePPI
CDK7	CDK7	P50613	*In vitro, in vivo*	HitPredict/WiKi-Pi/PIAN2/APID/PrePPI/PPI-finder
CEP170	CE170	Q5SW79	Proximity Label-MS	BioGIRD/APID/Mentha/PrePPI
CRK	CRK	Q63768	Two-hybrid	BioGIRD/IntAct/APID/Mentha/PrePPI
CXCR1	CXCR1	P25024	Co-immunoprecipitation	PubMed
CXCR2	CXCR2	P25025	Co-immunoprecipitation	PubMed
CXCR3	CXCR3	P49682	Co-immunoprecipitation	PubMed
CXCR4	CXCR4	P61073	Co-immunoprecipitation	PubMed
DAZAP2	DAZP2	Q15038	Two-hybrid	Mentha/PrePPI
DCTN1	DCTN1	Q14203	Proximity Label-MS	BioGIRD/APID/Mentha/PrePPI
DDB1	DDB1	Q16531	Co-fractionation	BioGIRD/APID/PrePPI
DNAAF2	KTU	Q9NVR5	Affinity Capture-MS	BioGIRD/APID/Mentha/PrePPI
DNM1	DYN1	Q05193	Pull down	IntAct/HitPredict/PIAN2/APID/Mentha
DNM2	DYN2	P50570	Pull down	IntAct/HitPredict/PIAN2/APID/PrePPI Mentha/PrePPI
EIF4B	IF4B	P23588	Co-fractionation	BioGIRD/APID/PrePPI
FANCC	FANCC	Q00597	Affinity Capture-MS/tandem affinity purification	BioGIRD/IntAct/PIAN2/Mentha
FBF1	FBF1	Q8TES7	Proximity Label-MS	BioGIRD/APID/Mentha/PrePPI
FHL3	FHL3	Q13643	Two-hybrid/two hybrid pooling approach	BioGIRD/IntAct/HitPredict/WiKi-Pi/PIAN2/APID/Mentha/PrePPI
FN1	FINC	P02751	Affinity Capture-MS	BioGIRD/HitPredict/PIAN2/APID/Mentha/PrePPI
FXR2	FXR2	P51116	Two-hybrid/two hybrid prey pooling approach	BioGIRD/IntAct/HitPredict/WiKi-Pi/APID/Mentha/PrePPI
FYTTD1	UIF	Q96QD9	Affinity Capture-MS	BioGIRD/APID/Mentha/PrePPI
GOLGA2	GOGA2	Q08379	Two-hybrid/two hybrid prey pooling approach	BioGIRD/IntAct/HitPredict/WiKi-Pi/APID/Mentha/PrePPI
HAX1	HAX1	O00165	Affinity Capture-MS	BioGIRD/APID/Mentha/PrePPI
HSPB1	HSPB1	P04792	Co-fractionation	BioGIRD/APID/Mentha/PrePPI
HSPE1	CH10	P61604	Co-fractionation	BioGIRD/APID/Mentha/PrePPI
ILK	ILK	Q13418	Affinity Capture-MS	BioGIRD/APID/Mentha/PrePPI
KLHL41	KRP1	O60662	Co-immunoprecipitation	PubMed
KRTAP4-2	KRA42	Q9BYR5	Two-hybrid/two hybrid prey pooling approach	BioGIRD/IntAct/HitPredict/WiKi-Pi/APID/Mentha/PrePPI
LGALS1	LEG1	P09382	Co-fractionation	BioGIRD/APID/Mentha/PrePPI
LPP	LPP	Q93052	Proximity ligation assay	IntAct/HitPredict/PIAN2/APID/Mentha/PrePPI/PPI-finder
LZTS2	LZTS2	Q9BRK4	Two-hybrid/two hybrid prey pooling approach	BioGIRD/IntAct/HitPredict/WiKi-Pi/APID/Mentha/PrePPI
MAP4	MAP4	P27816	Co-fractionation	BioGIRD/APID/Mentha/PrePPI
MDFI	MDFI	Q99750	Two-hybrid	BioGIRD/IntAct/HitPredict/WiKi-Pi/PIAN2/APID/Mentha/PrePPI
NFYA	NFYA	P23511	Affinity Capture-MS	BioGIRD/APID/Mentha/PrePPI
NXF1	NXF1	Q9UBU9	Co-fractionation	BioGIRD/APID/Mentha/PrePPI
OR10H3	OR10H3	O60404	Affinity Capture-MS	BioGIRD/APID/Mentha/PrePPI
PALLD	PALLD	Q8WX93	Co-immunoprecipitation/GSTpull-down	PubMed
PLSCR1	PLS1	O15162	Two-hybrid/two hybrid pooling approach	BioGIRD/IntAct/HitPredict/WiKi-Pi/PIAN2/APID/Mentha/PrePPI
POT1	POTE1	Q9NUX5	Two-hybrid/bimolecular fluorescence complementation	BioGIRD/IntAct/WiKi-Pi/PIAN2/APID/Mentha/PrePPI
PPP3CA	PP2BA	Q08209	phosphatase assay	IntAct/HitPredict/PIAN2/APID/Mentha
PRKACA	KAPCA	P17612	Biochemical Activity	BioGIRD/PIAN2/Mentha
PRKAR2B	KAP3	P31323	*In vitro, in vivo*	WiKi-Pi/PIAN2/APID
PRKCZ	KPCZ	P09217	Affinity Capture-MS	BioGIRD/Mentha
PRKG1	KGP1	Q13976	Biochemical Activity	BioGIRD/HitPredict/WiKi-Pi/PIAN2/APID/Mentha
PRKG2	KGP2	Q13237	*In vitro, in vivo*	HitPredict/WiKi-Pi/APID
PSMA3	PSA3	P25788	Two-hybrid/two hybrid prey pooling approach	BioGIRD/IntAct/HitPredict/WiKi-Pi/APID/Mentha
RBPMS	RBPMS	Q93062	Barcode fusion genetics two hybrid	IntAct/Mentha
RDH12	RDH12	Q96NR8	Affinity Capture-MS	BioGIRD/APID/Mentha/PrePPI
REL	REL	Q04864	Two-hybrid/two hybrid prey pooling approach	BioGIRD/IntAct/HitPredict/WiKi-Pi/APID/Mentha/PrePPI
RHOXF2	RHXF2	Q9BQY4	Two-hybrid/two hybrid prey pooling approach	BioGIRD/IntAct/HitPredict/WiKi-Pi/APID/Mentha/PrePPI
S100A11	S10AB	P31949	Co-immunoprecipitation	Pubmed/PPI-finder
SCLT1	SCLT1	Q96NL6	Proximity Label-MS	BioGIRD/APID/Mentha/PrePPI
SEPT3	SEPT3	Q9UH03	Two-hybrid/two hybrid prey pooling approach	BioGIRD/IntAct/HitPredict/WiKi-Pi/APID
SFN	1433S	P31947	Co-immunoprecipitation	Pubmed
SH2D2A	SH22A	Q9NP31	Affinity Capture-Luminescence/Two-hybrid	BioGIRD/IntAct/APID/Mentha/PrePPI
SLC25A32	MFTC	Q9H2D1	Affinity Capture-MS	BioGIRD/APID/Mentha/PrePPI
SLC25A44	S2544	Q96H78	Affinity Capture-MS	BioGIRD/APID/Mentha/PrePPI
SNRNP27	SNR27	Q8WVK2	Affinity Capture-MS	BioGIRD/APID
SPRY2	SPY2	O43597	Two-hybrid/two hybrid prey pooling approach	BioGIRD/IntAct/HitPredict/WiKi-Pi/APID/MenthaPrePPI
STAT3	STAT3	P40763	Two-hybrid	BioGIRD/IntAct/APID/Mentha/PrePPI
SUMO2	SUMO2	P61956	Affinity chromatography technology	BioGIRD/HitPredict/PrePPI
TAS2R41	T2R41	P59536	Affinity Capture-MS	BioGIRD/APID/Mentha/PrePPI
TCF4	ITF2	P15884	Two hybrid prey pooling approach/Two-hybrid	BioGIRD/IntAct/WiKi-Pi/APID/Mentha/PrePPI
TERF1	TERF1	P54274	Two-hybrid/bimolecular fluorescence complementation	BioGIRD/IntAct/WiKi-Pi/PIAN2/APID/Mentha/PrePPI
THAP1	THAP1	Q9NVV9	Two-hybrid/two hybrid prey pooling approach	BioGIRD/IntAct/HitPredict/WiKi-Pi/APID/Mentha/PrePPI
THAP8	THAP8	Q8NA92	Affinity Capture-MS	BioGIRD/APID/Mentha/PrePPI
TINF2	TINF2	Q9BSI4	Two-hybrid/bimolecular fluorescence complementation	BioGIRD/IntAct/HitPredict/PIAN2/APID/Mentha/PrePPI
TJP2	ZO2	Q9UDY2	Pull down	IntAct/HitPredict/PIAN2/APID/Mentha/PrePPI
TRIM27	TRI27	P14373	Two-hybrid/two hybrid prey pooling approach	BioGIRD/IntAct/HitPredict/WiKi-Pi/APID/Mentha/PrePPI
TRIP13	PCH2	Q15645	Two-hybrid	BioGIRD/WiKi-Pi/PIAN2/APID/Mentha/PrePPI
UBC	UBC	P0CG48	Affinity chromatography technology	HitPredict/BioGRID/PIAN2/PrePPI
VASP	VASP	P50552	Co-immunoprecipitation	PubMed/PrePPI
VIM	VIME	P08670	Co-immunoprecipitation/mass spectrometry	PubMed
ZBTB9	ZBTB9	Q96C00	Two-hybrid/two hybrid prey pooling approach	BioGIRD/IntAct/HitPredict/WiKi-Pi/APID/Mentha/PrePPI
ZC2HC1A	ZC21A	Q96GY0	Two-hybrid/two hybrid prey pooling approach	BioGIRD/IntAct/HitPredict/WiKi-Pi/Mentha
ZDHHC17	ZDH17	Q8IUH5	Affinity Capture-Western/Two-hybrid	BioGIRD/IntAct/HitPredict/PIAN2/APID/Mentha/PrePPI
ZNF764	ZN764	Q96H86	Affinity Capture-MS	BioGIRD/APID
ZYX	ZYX	Q15942	Reconstituted Complex/Two-hybrid/pull down	BioGIRD/IntAct/HitPredict/WiKi-Pi/PIAN2/APID/Mentha/PubMed/PrePPI/PPI-finder

**Table 2:** Cox regression analysis of LASP-1 interactors associated with survival of HBV-related HCC patients.

Covariates	Hazard radio	95% confidence interval	*P* value
LASP1	1.27	0.68–2.35	0.45
AHNAK	1.41	0.75–2.64	0.28
ARFGAP1	1.21	0.71–2.07	0.47
CDK7	0.80	0.53–1.21	0.29
CEP170	1.34	0.86–2.10	0.20
DAZAP2	1.51	0.70–3.24	0.29
DCTN1	0.96	0.62–1.50	0.86
DDB1	0.94	0.40–2.21	0.89
DNAAF2	1.08	0.65–1.78	0.77
FANCC	0.92	0.51–1.65	0.77
HAX1	1.59	0.94–2.69	0.08
HSPB1	0.91	0.59–1.41	0.68
HSPE1	0.82	0.48–1.39	0.47
ILK	0.55	0.31–0.97	0.04
MAP4	1.69	0.71–4.04	0.24
NFYA	2.60	1.20–5.64	0.02
PALLD	0.96	0.76–1.20	0.71
POT1	0.78	0.43–1.41	0.41
PSMA3	1.09	0.61–1.93	0.77
RBPMS	1.50	1.02–2.22	0.04
S100A11	1.04	0.81–1.35	0.75
SFN	1.03	0.91–1.16	0.67
SLC25A44	0.46	0.27–0.78	0.01
SNRNP27	0.47	0.22–0.99	0.04
SUMO2	0.82	0.36–1.85	0.63
TERF1	1.25	0.82–1.91	0.30
TRIM27	0.92	0.50–1.68	0.78
TRIP13	1.33	0.96–1.83	0.09
VASP	0.67	0.40–1.12	0.13
ZYX	0.75	0.45–1.26	0.28

**Table 3:** Cox regression analysis of LASP-1 interactors associated with recurrence of HBV-related HCC patients.

Covariates	Hazard radio	95% confidence interval	*P* value
LASP1	1.23	0.74–2.04	0.42
AHNAK	1.00	0.61–1.64	0.99
ARFGAP1	1.20	0.77–1.86	0.41
CDK7	0.92	0.66–1.30	0.65
CEP170	1.08	0.74–1.59	0.68
DAZAP2	1.28	0.68–2.41	0.45
DCTN1	0.94	0.65–1.36	0.76
DDB1	1.30	0.66–2.56	0.44
DNAAF2	0.94	0.62–1.41	0.76
FANCC	1.12	0.70–1.79	0.65
HAX1	1.48	0.93–2.35	0.10
HSPB1	0.95	0.68–1.34	0.77
HSPE1	1.09	0.70–1.68	0.71
ILK	0.81	0.51–1.30	0.39
MAP4	1.65	0.81–3.36	0.17
NFYA	1.58	0.83–3.02	0.16
PALLD	1.01	0.83–1.22	0.95
POT1	0.90	0.56–1.47	0.68
PSMA3	1.03	0.63–1.69	0.91
RBPMS	1.20	0.86–1.67	0.28
S100A11	1.02	0.82–1.27	0.83
SFN	1.06	0.96–1.17	0.28
SLC25A44	0.58	0.39–0.88	0.01
SNRNP27	0.45	0.24–0.83	0.01
SUMO2	0.98	0.49–1.96	0.95
TERF1	1.32	0.91–1.91	0.14
TRIM27	0.96	0.58–1.60	0.87
TRIP13	0.98	0.73–1.31	0.87
VASP	0.99	0.65–1.52	0.96
ZYX	0.68	0.44–1.05	0.08
