# Supplementary material for: Bioinformatics analysis of the proteins interacting with LASP-1 and their association with HBV-related hepatocellular carcinoma
Source: Sci Rep. 2017 Mar 7;7:44017. doi: 10.1038/srep44017 (PMC5339786; doi:10.1038/srep44017)
Supplement: Supplementary Information [file srep44017-s1.pdf]

Bioinformatics analysis of the proteins interacting with LASP-1 and their association with HBV-related hepatocellular carcinoma

Fan-Yun Kong, Ting Zhu, Nan Li, Yun-Fei Cai, Kai Zhou, Xiao Wei, Yan-Bo Kou, Hong-Juan You, Kui-Yang Zheng & Ren-Xian Tang

Supplementary Table S1. The information of predicated LASP-1 interactors without experimental validation

Uniprot Number	gene symbol	protein symbol	sources
Q8WWZ4	ABCA10	ABCAA	PPI-finder
P00519	ABL1	ABL1	PrePPI
O14639	ABLIM1	ABLM1	PPI-finder
Q16515	ACCN1	ASIC2	PPI-finder
O43707	ACTN4	ACTN4	PrePPI
O75077	ADAM23	ADA23	PPI-finder
Q8N157	AHI1	AHI1	PrePPI
P31749	AKT1	AKT1	PrePPI
Q92688	ANP32B	AN32B	PPI-finder
P05067	APP	A4	PrePPI
Q8IWW6	ARHGAP12	RHG12	PrePPI
Q68EM7	ARHGAP17	RHG17	PrePPI
Q6ZUM4	ARHGAP27	RHG27	PrePPI
A7KAX9	ARHGAP32	RHG32	PrePPI
A6NI28	ARHGAP42	RHG42	PrePPI
Q96DR7	ARHGEF26	ARHGQ	PrePPI
Q9NR80	ARHGEF4	ARHG4	PrePPI
Q15052	ARHGEF6	ARHG6	PPI-finder
**Continued**			

Uniprot Number	gene symbol	protein symbol	sources
O15143	ARPC1B	ARC1B	PrePPI
P32121	ARRB2	ARRB2	PrePPI
Q9ULH1	ASAP1	ASAP1	PrePPI
O15342	ATP6V0E1	ATP6V0E1	PrePPI
P61769	B2M	B2M	PrePPI
P56817	BACE1	BACE1	PrePPI
Q9Y5Z0	BACE2	BACE2	PrePPI
O95817	BAG3	BAG3	PPI-finder
P46379	BAG6	BAG6	PrePPI
P56945	BCAR1	BCAR1	PrePPI
O75815	BCAR3	BCAR3	PrePPI
O00193	C11orf58	SMAP	PPI-finder
Q9BWL3	C1orf43	CA043	PPI-finder
Q9BXJ3	C1QTNF4	C1QT4	PPI-finder
P54284	CACNB3		PrePPI
Q6IMN6	CAPRIN2	CAPR2	PPI-finder
O15234	CASC3	CASC3	PPI-finder
Q8WXE0	CASKIN2	CSKI2	PPI-finder
P22681	CBL	CBL	PPI-finder/PrePPI
P20963	CD247	CD3Z	PPI-finder
P10747	CD28	CD28	PPI-finder
P04234	CD3D	CD3D	PPI-finder
P07766	CD3E	CD3E	PPI-finder
B0YIY5	CD3G	B0YIY5	PPI-finder
P08962	CD63	CD63	PrePPI
P24941	CDK2	CDK2	PrePPI
P11802	CDK4	CDK4	PrePPI
P50750	CDK9	CDK9	PrePPI
Q00610	CLTC	CLTC	PrePPI
P23946	CMA1	CMA1	PrePPI
P46108	CRK	CRK	PrePPI
P46109	CRKL	CRKL	PrePPI
**Continued**			

Uniprot Number	gene symbol	protein symbol	sources
P41240	CSK	CSK	PrePPI
P48730	CSNK1D	KC1D	PrePPI
P68400	CSNK2A1	CSK21	PrePPI
P35222	CTNNB1	CTNB1	PrePPI
P07339	CTSD	CATD	PrePPI
P08311	CTSG	CATG	PrePPI
Q14247	CTTN	CTTN	PrePPI
Q14999	CUL7	CUL7	PrePPI
P98082	DAB2	DAB2	PrePPI
Q16643	DBN1	DREB	PPI-finder
Q9UJU6	DBNL	DBNL	PPI-finder/PrePPI
O00571	DDX3X	DDX3X	PPI-finder
Q14562	DHX8	DHX8	PPI-finder
Q15700	DLG2	DLG2	PrePPI
P11532	DMD	DMD	PPI-finder
Q14185	DOCK1	DOCK1	PrePPI
Q92608	DOCK2	DOCK2	PrePPI
Q8IZD9	DOCK3	DOCK3	PrePPI
Q8N1I0	DOCK4	DOCK4	PrePPI
Q9H7D0	DOCK5	DOCK5	PrePPI
Q99704	DOK1	DOK1	PPI-finder
O60496	DOK2	DOK2	PPI-finder
P00533	EGFR	EGFR	PrePPI
Q9HCK5	EIF2C4	AGO4	PPI-finder
Q15056	EIF4H	IF4H	PPI-finder
Q15717	ELAVL1	ELAVL1	PrePPI
Q92556	ELMO1	ELMO1	PrePPI
P50402	EMD	EMD	PPI-finder
Q09472	EP300	EP300	PrePPI
Q8TE68	EPS8L1	EPS8L1	PrePPI
Q9H6S3	EPS8L2	ES8L2	PrePPI
Q8TE67	EPS8L3	ES8L3	PrePPI
**Continued**			

Uniprot Number	gene symbol	protein symbol	sources
P04626	ERBB2	ERBB2	PrePPI
Q1RMC9	ERBB2IP	ERBB2IP	PPI-finder
P15311	EZR	EZRI	PrePPI
Q96CS3	FAF2	FAF2	PrePPI
Q96GY0	FAM164A		PrePPI
Q86WN1	FCHSD1	FCSD1	PrePPI
O94868	FCHSD2	FCSD2	PrePPI
Q13045	FLII	FLII	PrePPI
P21333	FLNA	FLNA	PrePPI
Q96RU3	FNBP1	FNBP1	PrePPI
Q5T0N5	FNBP1L	FBP1L	PrePPI
Q9BYC5	FUT8	FUT8	PrePPI
P06241	FYN	FYN	PrePPI
Q13283	G3BP1	G3BP1	PPI-finder
Q8WWW8	GAB3	GAB3	PrePPI
Q8NEA9	GMCL1L	GMCLL	PPI-finder
P04899	GNAI2	GNAI2	PrePPI
Q92917	GPKOW	GPKOW	PrePPI
P62993	GRB2	GRB2	PrePPI
P49841	GSK3B	GSK3B	PrePPI
P10144	GZMB	GRAB	PrePPI
P20718	GZMH	GRAH	PrePPI
P08631	HCK	HCK	PrePPI
P14317	HCLS1	HCLS1	PPI-finder
O95714	HERC2	HERC2	PrePPI
Q14103	HNRNPD	HNRPD	PPI-finder
P08238	HSP90AB1	HS90B	PPI-finder/PrePPI
P11021	HSPA5	GRP78	PrePPI
P50406	HTR6	5HT6R	PPI-finder
P42858	HTT	HD	PrePPI
O14920	IKBKB	IKKB	PrePPI
Q12906	ILF3	ILF3	PPI-finder
**Continued**			

Uniprot Number	gene symbol	protein symbol	sources
O15357	INPPL1	SHIP2	PrePPI
Q9Y283	INVS	INVS	PrePPI
P51617	IRAK1	IRAK1	PPI-finder
P05161	ISG15	ISG15	PrePPI
Q15811	ITSN1	ITSN1	PrePPI
Q9NZM3	ITSN2	ITSN2	PrePPI
P23458	JAK1	JAK1	PrePPI
Q8BW72	JMJD2A	KDM4A	PPI-finder
O60229	KALRN	KALRN	PrePPI
P22460	KCNA5	KCNA5	PrePPI
Q6NYC8	KIAA1949	PPR18	PrePPI
P06870	KLK1	KLK1	PrePPI
Q9Y337	KLK5	KLK5	PrePPI
P03952	KLKB1	KLKB1	PrePPI
Q14533	KRT81	KRT81	PPI-finder
Q53QV2	LBH	LBH	PPI-finder
P06239	LCK	LCK	PrePPI
Q13094	LCP2	LCP2	PPI-finder
P53667	LIMK1	LIMK1	PrePPI
O60711	LPXN	LPXN	PPI-finder
Q96AG4	LRRC59	LRC59	PrePPI
P07948	LYN	LYN	PrePPI
P61626	LYZ	LYSC	PrePPI
P28482	MAPK1	MK01	PPI-finder
P27361	MAPK3	MK03	PrePPI
Q9UQF2	MAPK8IP1	MAPK8IP1	PrePPI
Q9UIS9	MBD1	MBD1	PPI-finder
O15068	MCF2L	MCF2L	PrePPI
Q7L2J0	MEPCE	MEPCE	PrePPI
Q9Y4C4	MFHAS1	MFHA1	PPI-finder
Q96PC5	MIA2	MIA2	PrePPI
Q00013	MPP1	EM55	PrePPI
**Continued**			

Uniprot Number	gene symbol	protein symbol	sources
Q13368	MPP3	MPP3	PrePPI
P26038	MSN	MOES	PIPs/PrePPI
P35579	MYH9	MYH9	PPI-finder/PrePPI
O94832	MYO1D	MYO1D	PPI-finder
Q12965	MYO1E	MYO1E	PrePPI
Q6PIF6	MYO7B	MYO7B	PrePPI
Q14596	NBR1	NBR1	PrePPI
P14598	NCF1	NCF1	PrePPI
P16333	NCK1	NCK1	PrePPI
O43639	NCK2	NCK2	PrePPI
Q9NZQ3	NCKIPSD	SPN90	PrePPI
Q9HCD5	NCOA5	NCOA5	PPI-finder
O43181	NDUFS4	NDUS4	PPI-finder
O76041	NEBL	NEBL	PPI-finder
P46934	NEDD4	NEDD4	PrePPI
Q15843	NEDD8	NEDD8	PrePPI
Q14511	NEDD9	CASL	PrePPI
Q99519	NEU1	NEUR1	PPI-finder
Q00653	NFKB2	NFKB2	PrePPI
Q8IVI9	NOSTRIN	NOSTN	PrePPI
P46531	NOTCH1	NOTC1	PrePPI
O15259	NPHP1	NPHP1	PrePPI
Q8TAT6	NPLOC4	NPL4	PrePPI
P06748	NPM1	NPM	PrePPI
P04150	NR3C1	GCR	PrePPI
Q9Y5A7	NUB1	NUB1	PrePPI
Q6UXH9	PAMR1	PAMR1	PrePPI
Q86U86	PBRM1	PB1	PPI-finder
Q15366	PCBP2	PCBP2	PPI-finder
Q9NR12	PDLIM7	PDLI7	PrePPI
Q6ZUJ8	PIK3AP1	BCAP	PrePPI
P27986	PIK3R1	P85A	PrePPI
**Continued**			

Uniprot Number	gene symbol	protein symbol	sources
O00459	PIK3R2	P85B	PrePPI
Q99569	PKP4	PKP4	PPI-finder
P16885	PLCG2	PLCG2	PrePPI
Q96BZ4	PLD4	PLD4	PPI-finder
P13797	PLS3	PLST	PrePPI
Q13131	PRKAA1	AAPK1	PrePPI
P22694	PRKACB	KAPCB	PrePPI
P22891	PROZ	PROZ	PrePPI
P07478	PRSS2	TRY2	PrePPI
Q16651	PRSS8	PRSS8	PrePPI
P24158	PRTN3	PRTN3	PrePPI
Q15185	PTGES3	TEBP	PPI-finder
Q06124	PTPN11	PTN11	PPI-finder
Q99952	PTPN18	PTN18	PPI-finder/PrePPI
P49023	PXN	PAXI	PPI-finder/PrePPI
P54727	RAD23B	RD23B	PrePPI
P52306	RAP1GDS1	GDS1	PrePPI
P20936	RASA1	RASA1	PrePPI
Q9BYM8	RBCK1	HOIL1	PrePPI
O60673	REV3L	DPOLZ	PPI-finder
Q9UFD9	RIMBP3	RIM3A	PrePPI
A6NNM3	RIMBP3B	RIM3B	PrePPI
A6NJZ7	RIMBP3C	RIM3C	PrePPI
Q8TB24	RIN3	RIN3	PrePPI
Q13546	RIPK1	RIPK1	PrePPI
P50914	RPL14	RL14	PrePPI
Q02878	RPL6	RL6	PrePPI
P05386	RPLP1	RLA1	PPI-finder
P62979	RPS27A	RS27A	PrePPI
P62701	RPS4X	RS4X	PrePPI
Q15418	RPS6KA1	KS6A1	PrePPI
Q9BVN2	RUSC1	RUSC1	PrePPI
**Continued**			

Uniprot Number	gene symbol	protein symbol	sources
Q8N2Y8	RUSC2	RUSC2	PrePPI
Q9NSI8	SAMSN1	SAMN1	PrePPI
O94885	SASH1	SASH1	PrePPI
O75995	SASH3	SASH3	PrePPI
Q15436	SEC23A	SC23A	PrePPI
O95487	SEC24B	SC24B	PPI-finder
Q86TU7	SETD3	SETD3	PPI-finder
Q9C0A6	SETD5	SETD5	PrePPI
P23246	SFPQ	SFPQ	PrePPI
Q7Z4S9	SH2D6	SH2D6	PrePPI
Q9P0V3	SH3BP4	SH3B4	PrePPI
Q5HYK7	SH3D19	SH319	PrePPI
A4FU49	SH3D21	SH321	PrePPI
Q99961	SH3GL1	SH3G1	PrePPI
Q99962	SH3GL2	SH3G2	PrePPI
Q9NR46	SH3GLB2	SHLB2	PrePPI
Q96B97	SH3KBP1	SH3K1	PrePPI
Q7Z6J0	SH3RF1	SH3R1	PrePPI
Q8TEC5	SH3RF2	SH3R2	PrePPI
Q8TEJ3	SH3RF3	SH3R3	PrePPI
Q8TE82	SH3TC1	S3TC1	PrePPI
Q96HL8	SH3YL1	SH3Y1	PrePPI
Q9UPX8	SHANK2	SHAN2	PrePPI
P29353	SHC1	SHC1	PrePPI
Q5VZ18	SHE		PrePPI
Q8TBC3	SHKBP1	SHKB1	PrePPI
Q9Y3P8	SIT1	SIT1	PPI-finder
Q13239	SLA	SLAP1	PrePPI
O14745	SLC9A3R1	NHRF1	PrePPI
P84022	SMAD3	SMAD3	PrePPI
Q96RF0	SNX18	SNX18	PrePPI
Q8WV41	SNX33	SNX33	PrePPI
**Continued**			

Uniprot Number	gene symbol	protein symbol	sources
Q9Y5X1	SNX9	SNX9	PrePPI
Q9BX66	SORBS1	SRBS1	PrePPI
Q8IXA5	SPACA3	SACA3	PrePPI
Q8IWB4	SPATA31A7	S31A7	PPI-finder
P02549	SPTA1	SPTA1	PrePPI
Q13813	SPTAN1	SPTN1	PrePPI
Q13501	SQSTM1	SQSTM	PrePPI
P12931	SRC	SRC	PrePPI
Q6ZMT1	STAC2	STAC2	PrePPI
Q96MF2	STAC3	STAC3	PrePPI
Q92783	STAM	STAM1	PrePPI
O75886	STAM2	STAM2	PrePPI
Q14849	STARD3	STAR3	PPI-finder
P42224	STAT1	STAT1	PrePPI
Q13586	STIM1	STIM1	PrePPI
P16949	STMN1	STMN1	PPI-finder
P63165	SUMO1	SUMO1	PrePPI
O00267	SUPT5H	SPT5H	PrePPI
Q7KZ85	SUPT6H	SPT6H	PrePPI
P26639	TARS	SYTC	PrePPI
Q13488	TCIRG1	VPP3	PrePPI
P48775	TDO2 TDO	T23O	PPI-finder
O14746	TERT	TERT	PPI-finder
Q92734	TFG	TFG	PrePPI
P31483	TIA1	TIA1	PPI-finder
P04183	TK1	KITH	PrePPI
Q9Y490	TLN1	TLN1	PrePPI
Q6NUQ4	TMEM214	TM214	PrePPI
O95807	TMEM50A	TM50A	PPI-finder
Q5T4D3	TMTC4	TMTC4	PPI-finder
Q07912	TNK2	ACK1	PrePPI
P04637	TP53	P53	PrePPI
**Continued**			

Uniprot Number	gene symbol	protein symbol	sources
Q12888	TP53BP1	TP53B	PPI-finder
Q13625	TP53BP2	ASPP2	PrePPI
Q9BUZ4	TRAF4	TRAF4	PPI-finder
Q13263	TRIM28	TIF1B	PrePPI
Q9HCM9	TRIM39	TRI39	PPI-finder
Q9BZR9	TRIM8	TRIM8	PrePPI
O75962	TRIO	TRIO	PPI-finder
Q15642	TRIP10	CIP4	PrePPI
Q9C0H2	TTYH3	TTYH3	PrePPI
Q14157	UBAP2L	UBP2L	PPI-finder
P0CG47	UBB	UBB	PrePPI
O15205	UBD	UBD	PrePPI
O14562	UBFD1	UBFD1	PrePPI
Q9UHD9	UBQLN2	UBQL2	PrePPI
Q9NRR5	UBQLN4	UBQL4	PrePPI
Q96T88	UHRF1	UHRF1	PrePPI
Q9C0B0	UNK	UNK	PPI-finder
P51784	USP11	UBP11	PrePPI
P52735	VAV2	VAV2	PrePPI
P18206	VCL	VINC	PrePPI
P55072	VCP	TERA	PrePPI
Q969T9	WBP2	WBP2	PPI-finder
P07947	YES1	YES	PPI-finder/PrePPI
P31946	YWHAB	1433B	PrePPI
P63104	YWHAZ	1433Z	PrePPI
P43403	ZAP70	ZAP70	PPI-finder
Q86UK7	ZNF598	ZN598	PPI-finder

Supplementary Table S2. The details of protein class of LASP-1 interactors identified by PANTHER classification system.

Category	Term	Count	%	Genes
PANTHER-Protein class	calcium-binding protein (PC00060)	6	5.50%	SLC25A32,PRKCZ,PRKACA,slc25a44,PPP3CA,S100A11
PANTHER-Protein class	cell adhesion molecule (PC00069)	7	6.40%	LGALS1,PALLD, CXCR2,CXCR4,CXCR1, CD81,CXCR3
PANTHER-Protein class	cell junction protein (PC00070)	4	3.70%	LPP,ZYX,SCLT1,TJP2
PANTHER-Protein class	chaperone (PC00072)	3	2.80%	SFN,HSPB1,HSPE1
PANTHER-Protein class	cytoskeletal protein (PC00085)	12	11.00%	DNM2,ZBTB9,SEPT3,LPP,DCTN1,ACTB,ZYX,DNM1, ACTC1,SCLT1,ACTA1,VIM
PANTHER-Protein class	defense/immunity protein (PC00090)	6	5.50%	PALLD,CCDC8, CXCR2,CXCR4,CXCR1,CXCR3
PANTHER-Protein class	enzyme modulator (PC00095)	8	7.30%	DNM2, SEPT3, LPP,ZYX,ARFGAP1, ARFGAP1, DNM1,SCLT1
PANTHER-Protein class	hydrolase (PC00121)	10	9.20%	DNM2, ZBTB9,PPP3CA, PALLD, CXCR2,CXCR4, CXCR1,DNM1,SFN,CXCR3
PANTHER-Protein class	membrane traffic protein (PC00150)	1	0.90%	GOLGA2
PANTHER-Protein class	nucleic acid binding (PC00171)	20	18.30%	THAP8,POT1,TCF4,SLC25A32,DDB1,TERF1,RHOXF2, slc25a44, REL,ARFGAP1,STAT3,THAP1, NXF1, FXR2,UBC,ARFGAP1,RBPMS,EIF4B, SFN,NFYA
PANTHER-Protein class	oxidoreductase (PC00176)	1	0.90%	RDH12
PANTHER-Protein class	receptor (PC00197)	6	5.50%	OR10H3,CXCR2,CXCR4, CXCR1,CD81, CXCR3
PANTHER-Protein class	signaling molecule (PC00207)	5	4.60%	OR10H3,CXCR2,CXCR4, CXCR1,CD81, CXCR3
PANTHER-Protein class	structural protein (PC00211)	1	0.90%	VIM
PANTHER-Protein class	transcription factor (PC00218)	8	7.30%	TCF4, RHOXF2,ZNF764, REL,STAT3,SPRY2,FHL3, NFYA
PANTHER-Protein class	transfer/carrier protein (PC00219)	5	4.60%	SLC25A32,PRKCZ,PRKACA,slc25a44,PLSCR1
PANTHER-Protein class	transferase (PC00220)	4	3.70%	PRKCZ, PRKACA,ILK,CDK7
PANTHER-Protein class	transporter (PC00227)	2	1.80%	SLC25A32,slc25a44

Supplementary Table S3. The details of protein location of LASP-1 interactors identified by UniProtKB database.

Category	Term	Count	%	Gene
UniProt-Protein location	Cytoplasm	47	26.25	ZYX,VIM,VASP,UBC,TRIM27,TERF1,STAT3,SPRY2,SH2D2A,SFN, SEPT3,SCLT1,S100A11,RBPMS,PSMA3,PRKG1,PRKCZ,PRKAR2B, PRKACA,PALLD,NXF1,MDFI,MAP4,LZTS2,LPP,KLHL41,ILK,HSPB1, GOLGA2,FXR2,FBF1,FANCC,DNM2,DNM1,DNAAF2,DDB1,DCTN1, DAZAP2,CRK,CEP170,CDK7,CCDC8,ATXN1,ARFGAP1,ACTC1,ACTB, ACTA1
UniProt-Protein location	cytoskeleton	22	12.29	ZYX,VASP,TERF1,SPRY2,SEPT3,SCLT1,PRKAR2B,PALLD,MAP4, LZTS2,KLHL41,HSPB1,GOLGA2,FBF1,DNM2,DNM1,DCTN1,CEP170, CCDC8,ACTC1,ACTB,ACTA1
UniProt-Protein location	Nucleus	37	20.67	ZYX,ZNF764,ZBTB9,UBC,TRIP13,TRIM27,TINF2,THAP1,TERF1,TCF4, SUMO2,STAT3,SNRNP27,SFN,S100A11,RHOXF2,REL,RBPMS,PSMA3, PRKACA,PPP3CA,POT1,PLSCR1,NXF1,NFYA,MDFI,LPP,HSPB1, FYTTD1,FHL3,FANCC,DDB1,DAZAP2,CDK7,ATXN1,AHNAK,ACD
UniProt-Protein location	Membrane	16	8.94	TJP2,TAS2R41,PRKG2,PRKACA,PPP3CA,PLSCR1,OR10H3,LPP,ILK, DNM2,CXCR1,CXCR2,CXCR3,CXCR4,CRK,CD81
UniProt-Protein location	Multi-pass membrane protein	10	5.59	ZDHHC17,TAS2R41,slc25a44,SLC25A32,OR10H3,CXCR1,CXCR2, CXCR3,CXCR4,CD81
UniProt-Protein location	Secreted	3	1.68	SFN,LGALS1,FN1
UniProt-Protein location	Chromosome	4	2.23	TINF2,TERF1,POT1,ACD
UniProt-Protein location	telomere	4	2.23	TINF2,TERF1,POT1,ACD
UniProt-Protein location	Mitochondrion	5	2.79	slc25a44,SLC25A32,PRKACA,HSPE1,HAX1
UniProt-Protein location	Cell junction	11	6.15	ZYX,ZDHHC17,VASP,TJP2,TINF2,SEPT3,PRKCZ,PALLD,LPP,ILK, FBF1
UniProt-Protein location	focal adhesion	5	2.79	FHL3,ILK,PALLD,VASP,ZYX
UniProt-Protein location	Golgi apparatus	3	1.68	ZDHHC17,GOLGA2,ARFGAP1
UniProt-Protein location	spindle	3	1.68	TERF1,HSPB1,CEP170
UniProt-Protein location	Lipid-anchor	3	1.68	PRKG2,PRKACA,PLSCR1
UniProt-Protein location	centrosome	6	3.35	SCLT1,LZTS2,FBF1,DCTN1,CEP170,CCDC8

Supplementary Table S4. The details of GO terms of LASP-1 interactors.

**Category**	**Term**	**Count**	**%**	**P Value**	**Genes**
GOTERM_BP_DIRECT	GO:0007165~signal transduction	14	6.45	0.001	PRKCZ, LGALS1, S100A11, CXCR2, SFN, PRKG2, PRKG1, STAT3, SH2D2A, PRKAR2B, ZDHHC17, PRKACA, ZYX, DNM2
GOTERM_BP_DIRECT	GO:0045944~positive regulation of transcription from RNA polymerase II promoter	13	6.00	0.003	ATXN1, SUMO2, PLSCR1, REL, HAX1, CD81, UBC, CXCR3, PPP3CA, NFYA, CDK7, TCF4, STAT3
GOTERM_BP_DIRECT	GO:0006915~apoptotic process	10	4.61	0.003	PLSCR1, ACTC1, CXCR4, LGALS1, VIM, PSMA3, UBC, CXCR3, SFN, TJP2
GOTERM_BP_DIRECT	GO:0043066~negative regulation of apoptotic process	10	4.61	6.59E-04	SPRY2, PRKCZ, ACTC1, HAX1, DDB1, PSMA3, UBC, HSPB1, STAT3, FN1
GOTERM_BP_DIRECT	GO:0016032~viral process	9	4.15	0.005	CXCR4, DDB1, VIM, PSMA3, UBC, NXF1, CDK7, ZYX, STAT3
GOTERM_BP_DIRECT	GO:0007596~blood coagulation	8	3.69	0.010	ACTB, PRKAR2B, PRKCZ, PRKACA, PRKG2, PRKG1, CRK, FN1
GOTERM_BP_DIRECT	GO:0000278~mitotic cell cycle	7	3.23	0.019	PRKAR2B, PSMA3, UBC, PRKACA, CDK7, DCTN1, GOLGA2
GOTERM_BP_DIRECT	GO:0048010~vascular endothelial growth factor receptor signaling pathway	7	3.23	0.003	ACTB, SH2D2A, PRKCZ, PSMA3, UBC, HSPB1, CRK
GOTERM_BP_DIRECT	GO:0061024~membrane organization	7	3.23	0.002	ACTB, ARFGAP1, UBC, SFN, DCTN1, DNM2, GOLGA2
GOTERM_BP_DIRECT	GO:0000086~G2/M transition of mitotic cell cycle	7	3.23	6.64E-05	PRKAR2B, UBC, PRKACA, CDK7, DCTN1, DNM2, TERF1
GOTERM_BP_DIRECT	GO:0048011~neurotrophin TRK receptor signaling pathway	6	2.76	0.046	PRKAR2B, PSMA3, UBC, PRKACA, CRK, STAT3
GOTERM_BP_DIRECT	GO:0008283~cell proliferation	6	2.76	0.034	SH2D2A, ILK, CD81, TRIM27, CDK7, STAT3
GOTERM_BP_DIRECT	GO:0006954~inflammatory response	6	2.76	0.033	PRKCZ, REL, CXCR4, CXCR1, CXCR2, CXCR3
GOTERM_BP_DIRECT	GO:0010628~positive regulation of gene expression	6	2.76	0.006	SPRY2, PLSCR1, ACTC1, ACTA1, VIM, FN1
GOTERM_BP_DIRECT	GO:0012501~programmed cell death	5	2.30	0.008	VIM, PSMA3, UBC, SFN, TJP2
GOTERM_BP_DIRECT	GO:0006289~nucleotide-excisi on repair	5	2.30	0.002	SUMO2, DDB1, UBC, CDK7, FANCC
GOTERM_BP_DIRECT	GO:0006928~movement of cell or subcellular component	5	2.30	8.60E-04	ACTB, VIM, HSPB1, CXCR3, STAT3

Continued

Category	Term	Count	%	P Value	Genes
GOTERM_BP_DIRECT	GO:0043123~positive regulation of I-kappaB kinase/NF-kappaB signaling	4	1.84	0.041	ZDHHC17, REL, LGALS1, UBC
GOTERM_BP_DIRECT	GO:0000082~G1/S transition of mitotic cell cycle	4	1.84	0.039	PSMA3, UBC, PPP3CA, CDK7
GOTERM_BP_DIRECT	GO:0002223~stimulatory C-type lectin receptor signaling pathway	4	1.84	0.023	PSMA3, UBC, PRKACA, PPP3CA
GOTERM_BP_DIRECT	GO:0006469~negative regulation of protein kinase activity	4	1.84	0.009	ILK, TRIM27, HSPB1, SFN
GOTERM_BP_DIRECT	GO:0070098~chemokine-medi ated signaling pathway	4	1.84	0.005	CXCR4, CXCR1, CXCR2, CXCR3
GOTERM_BP_DIRECT	GO:0000723~telomere maintenance	4	1.84	0.004	ACD, TINF2, POT1, TERF1
GOTERM_BP_DIRECT	GO:0070911~global genome nucleotide-excision repair	4	1.84	0.004	SUMO2, DDB1, UBC, CDK7
GOTERM_BP_DIRECT	GO:0071333~cellular response to glucose stimulus	4	1.84	0.003	LGALS1, PRKACA, PPP3CA, FN1
GOTERM_BP_DIRECT	GO:0031623~receptor internalization	4	1.84	8.63E-04	CD81, CXCR1, CXCR2, DNM2
GOTERM_BP_DIRECT	GO:0038061~NIK/NF-kappaB signaling	3	1.38	0.043	REL, PSMA3, UBC
GOTERM_BP_DIRECT	GO:0006953~acute-phase response	3	1.38	0.017	PLSCR1, STAT3, FN1
GOTERM_BP_DIRECT	GO:0070527~platelet aggregation	3	1.38	0.017	ACTB, ILK, HSPB1
GOTERM_BP_DIRECT	GO:0030049~muscle filament sliding	3	1.38	0.015	ACTC1, ACTA1, VIM
GOTERM_BP_DIRECT	GO:0048741~skeletal muscle fiber development	3	1.38	0.009	ACTA1, PPP3CA, KLHL41
GOTERM_BP_DIRECT	GO:0007004~telomere maintenance via telomerase	3	1.38	0.003	ACD, POT1, TERF1
GOTERM_BP_DIRECT	GO:0051974~negative regulation of telomerase activity	3	1.38	0.002	TINF2, POT1, TERF1
GOTERM_BP_DIRECT	GO:0002407~dendritic cell chemotaxis	3	1.38	0.002	CXCR4, CXCR1, CXCR2
GOTERM_BP_DIRECT	GO:0032211~negative regulation of telomere maintenance via telomerase	3	1.38	0.002	ACD, TINF2, TERF1

Continued

Category	Term	Count	%	P Value	Genes
GOTERM_BP_DIRECT	GO:0032202~telomere assembly	3	1.38	7.19E-05	ACD, TINF2, POT1
GOTERM_BP_DIRECT	GO:0003334~keratinocyte development	2	0.92	0.048	SFN, PALLD
GOTERM_BP_DIRECT	GO:0042119~neutrophil activation	2	0.92	0.048	CXCR4, CXCR2
GOTERM_BP_DIRECT	GO:0070198~protein localization to chromosome, telomeric region	2	0.92	0.044	ACD, TINF2
GOTERM_BP_DIRECT	GO:0051168~nuclear export	2	0.92	0.039	ATXN1, LZTS2
GOTERM_BP_DIRECT	GO:0070200~establishment of protein localization to telomere	2	0.92	0.030	ACD, POT1
GOTERM_BP_DIRECT	GO:0035020~regulation of Rac protein signal transduction	2	0.92	0.029	CRK, DNM2
GOTERM_BP_DIRECT	GO:0030240~skeletal muscle thin filament assembly	2	0.92	0.024	ACTC1, ACTA1
GOTERM_BP_DIRECT	GO:0016233~telomere capping	2	0.92	0.020	ACD, POT1
GOTERM_BP_DIRECT	GO:0090131~mesenchyme migration	2	0.92	0.020	ACTC1, ACTA1
GOTERM_BP_DIRECT	GO:0038112~interleukin-8-mediated signaling pathway	2	0.92	0.010	CXCR1, CXCR2
GOTERM_CC_DIRECT	GO:0005737~cytoplasm	46	15.49	2.50E-07	PRKCZ, SEPT3, LZTS2, VIM, CXCR3, SFN, PRKG1, DAZAP2, PRKAR2B, ACD, CXCR4, DNAAF2, CEP170, ILK, ZYX, PPP3CA, KLHL41, AHNAK, TERF1, FANCC, ACTB, MDFI, ACTC1, LPP, DDB1, LGALS1, TRIM27, S100A11, CDK7, ARHGEF15, FXR2, PALLD, STAT3, VASP, DCTN1, ATXN1, SH2D2A, RBPMS, ZDHHC17, PSMA3, HSPB1, MAP4, CRK, TJP2, CCDC8, DNM2
GOTERM_CC_DIRECT	GO:0005634~nucleus	41	13.80	1.73E-04	ZBTB9, FHL3, NFYA, SFN, DAZAP2, POT1, SUMO2, SPRY2, ACD, REL, THAP1, PRKACA, ZYX, KLHL41, TCF4, TINF2, RHOXF2, AHNAK, TRIP13, GOLGA2, FANCC, TERF1, MDFI, LPP, DDB1, LGALS1, TRIM27, S100A11, NXF1, CDK7, FXR2, PALLD, STAT3, ATXN1, PLSCR1, PSMA3, UBC, HSPB1, ZNF764, CRK, DNM2

Continued

Category	Term	Count	%	P Value	Genes
GOTERM_CC_DIRECT	GO:0005829~cytosol	35	11.78	1.47E-06	ARFGAP1, PRKCZ, VIM, CXCR2, CXCR3, PRKG2, SFN, PRKG1, FBF1, PRKAR2B, SPRY2, REL, CXCR4, ILK, PRKACA, PPP3CA, AHNAK, FANCC, ACTB, SCLT1, ACTC1, ACTA1, NXF1, DCTN1, VASP, STAT3, EIF4B, PLSCR1, SH2D2A, PSMA3, UBC, HSPB1, TJP2, CRK, DNM2
GOTERM_CC_DIRECT	GO:0005886~plasma membrane	30	10.10	0.005	PRKCZ, VIM, CXCR1, CXCR2, CXCR3, PRKG1, PRKAR2B, SPRY2, CXCR4, ILK, KLHL41, ZYX, AHNAK, TAS2R41, ACTB, LPP, PALLD, OR10H3, VASP, STAT3, PLSCR1, CD81, UBC, MAP4, HSPB1, TJP2, CRK, DNM1, DNM2, CCDC8
GOTERM_CC_DIRECT	GO:0070062~extracellular exosome	27	9.09	3.22E-04	PRKCZ, VIM, SFN, PRKAR2B, CXCR4, PRKACA, HSPE1, AHNAK, FN1, ACTB, SCLT1, ACTC1, ACTA1, DDB1, LGALS1, S100A11, FXR2, VASP, PLSCR1, CD81, PSMA3, UBC, MAP4, HSPB1, CRK, DNM1, DNM2
GOTERM_CC_DIRECT	GO:0005654~nucleoplasm	24	8.08	0.001	ACTB, DDB1, TRIM27, NXF1, CDK7, NFYA, STAT3, POT1, ATXN1, SUMO2, RBPMS, ACD, REL, FYTTD1, PSMA3, ILK, UBC, PRKACA, PPP3CA, SNRNP27, TJP2, TINF2, FANCC, TERF1
GOTERM_CC_DIRECT	GO:0005925~focal adhesion	13	4.38	3.08E-07	ACTB, ACTC1, LPP, CD81, VIM, ILK, FHL3, HSPB1, ZYX, PALLD, VASP, AHNAK, DNM2
GOTERM_CC_DIRECT	GO:0005813~centrosome	9	3.03	7.98E-04	PRKAR2B, SCLT1, LZTS2, CEP170, PRKACA, DCTN1, FBF1, DNM2, CCDC8
GOTERM_CC_DIRECT	GO:0005874~microtubule	8	2.69	4.84E-04	SPRY2, LZTS2, CEP170, MAP4, DNM1, DCTN1, DNM2, GOLGA2
GOTERM_CC_DIRECT	GO:0005856~cytoskeleton	6	2.02	0.025	ACTB, SPRY2, SEPT3, VIM, HSPB1, KLHL41
GOTERM_CC_DIRECT	GO:0015629~actin cytoskeleton	6	2.02	0.004	ACTA1, HAX1, PALLD, CRK, VASP, AHNAK
GOTERM_CC_DIRECT	GO:0030027~lamellipodium	6	2.02	9.05E-04	ACTC1, ACTA1, HAX1, ILK, PALLD, DNM2
GOTERM_CC_DIRECT	GO:0000784~nuclear chromosome, telomeric region	5	1.68	0.003	ACD, DDB1, TINF2, POT1, TERF1
GOTERM_CC_DIRECT	GO:0031012~extracellular matrix	4	1.35	0.047	PLSCR1, LGALS1, ILK, FN1
**Continued**					

Category	Term	Count	%	P Value	Genes
GOTERM_CC_DIRECT	GO:0072562~blood microparticle	4	1.35	0.030	ACTB, ACTC1, ACTA1, FN1
GOTERM_CC_DIRECT	GO:0030018~Z disc	4	1.35	0.014	FHL3, HSPB1, PPP3CA, PALLD
GOTERM_CC_DIRECT	GO:0001725~stress fiber	4	1.35	0.002	ACTA1, ILK, FHL3, ZYX
GOTERM_CC_DIRECT	GO:0031252~cell leading edge	4	1.35	8.33E-04	PRKCZ, CXCR4, VIM, DCTN1
GOTERM_CC_DIRECT	GO:0000783~nuclear telomere cap complex	4	1.35	8.48E-06	ACD, TINF2, POT1, TERF1
GOTERM_CC_DIRECT	GO:0070187~telosome	4	1.35	3.56E-06	ACD, TINF2, POT1, TERF1
GOTERM_CC_DIRECT	GO:0005884~actin filament	3	1.01	0.025	ACTC1, ACTA1, PALLD
GOTERM_CC_DIRECT	GO:0000781~chromosome, telomeric region	3	1.01	0.018	TINF2, POT1, TERF1
GOTERM_CC_DIRECT	GO:0030017~sarcomere	3	1.01	0.014	ACTC1, ACTA1, ILK
GOTERM_CC_DIRECT	GO:0005952~cAMP-dependent protein kinase complex	2	0.67	0.037	PRKAR2B, PRKACA
GOTERM_CC_DIRECT	GO:0097539~ciliary transition fiber	2	0.67	0.028	SCLT1, FBF1
GOTERM_MF_DIRECT	GO:0005515~protein binding	76	49.03	1.09E-11	SEPT3, LZTS2, KRTAP4-2, CXCR2, PRKG1, SPRY2, CXCR4, ILK, PRKACA, ZYX, AHNAK, GOLGA2, FANCC, ACTA1, DDB1, ARHGEF15, CDK7, PALLD, VASP, DCTN1, SH2D2A, RBPMS, ZDHHC17, PSMA3, CD81, UBC, HSPB1, ARFGAP1, THAP8, C11ORF65, PRKCZ, HAX1, ZBTB9, VIM, FHL3, SFN, NFYA, DAZAP2, FBF1, POT1, SUMO2, ACD, REL, DNAAF2, CEP170, THAP1, HSPE1, PPP3CA, KLHL41, TCF4, RHOXF2, TINF2, TRIP13, TERF1, FN1, MDFI, ACTB, LPP, LGALS1, S100A11, TRIM27, NXF1, FXR2, STAT3, RDH12, ATXN1, EIF4B, PLSCR1, ZC2HC1A, FYTTD1, MAP4, SNRNP27, CRK, TJP2, DNM1, DNM2
GOTERM_MF_DIRECT	GO:0044822~poly(A) RNA binding	14	9.03	0.005	LGALS1, NXF1, FXR2, EIF4B, SUMO2, RBPMS, FYTTD1, UBC, HSPB1, MAP4, HSPE1, ZYX, DNM1, AHNAK
GOTERM_MF_DIRECT	GO:0042802~identical protein binding	11	7.10	0.001	ACTB, ATXN1, ZDHHC17, VIM, HSPB1, THAP1, TCF4, FXR2, DNM1, STAT3, TRIP13
GOTERM_MF_DIRECT	GO:0019901~protein kinase binding	10	6.45	1.71E-04	SPRY2, PRKCZ, ILK, HSPB1, PRKACA, SFN, DNM1, STAT3, DNM2, GOLGA2
GOTERM_MF_DIRECT	GO:0008022~protein C-terminus binding	7	4.52	4.61E-04	ATXN1, SCLT1, VIM, CDK7, TCF4, TJP2, DNM1
GOTERM_MF_DIRECT	GO:0004871~signal transducer activity	5	3.23	0.019	ZDHHC17, LGALS1, ILK, CXCR2, STAT3
**Continued**					

Category	Term	Count	%	P Value	Genes
GOTERM_MF_DIRECT	GO:0017124~SH3 domain binding	5	3.23	0.003	PLSCR1, SH2D2A, ILK, VASP, DNM2
GOTERM_MF_DIRECT	GO:0042162~telomeric DNA binding	4	2.58	2.56E-04	ACD, TINF2, POT1, TERF1
GOTERM_MF_DIRECT	GO:0043130~ubiquitin binding	3	1.94	0.0415804	CXCR4, HSPB1, TERF1
GOTERM_MF_DIRECT	GO:0050998~nitric-oxide synthase binding	3	1.94	0.004	ACTB, DNM1, DNM2
GOTERM_MF_DIRECT	GO:0016494~C-X-C chemokine receptor activity	3	1.94	5.56E-04	CXCR4, CXCR2, CXCR3
GOTERM_MF_DIRECT	GO:0010521~telomerase inhibitor activity	2	1.29	0.041	TINF2, POT1
GOTERM_MF_DIRECT	GO:0098505~G-rich strand telomeric DNA binding	2	1.29	0.036	POT1, TERF1
GOTERM_MF_DIRECT	GO:0031749~D2 dopamine receptor binding	2	1.29	0.016	DNM1, DNM2
GOTERM_MF_DIRECT	GO:0019959~interleukin-8 binding	2	1.29	0.016	CXCR1, CXCR2
GOTERM_MF_DIRECT	GO:0008426~protein kinase C inhibitor activity	2	1.29	0.016	HSPB1, SFN
GOTERM_MF_DIRECT	GO:0004692~cGMP-dependen t protein kinase activity	2	1.29	0.0106	PRKG2, PRKG1
GOTERM_MF_DIRECT	GO:0004918~interleukin-8 receptor activity	2	1.29	0.0106	CXCR1, CXCR2

Supplementary Table S5. The results of KEGG pathway analysis of LASP-1 interactors.

Category	Term	Count	%	P Value	Genes
KEGG_PATHWAY	hsa04062:Chemokine signaling pathway	8	16	1.48E-04	PRKCZ, CXCR4, CXCR1, CXCR2, PRKACA, CXCR3, CRK, STAT3
KEGG_PATHWAY	hsa04144:Endocytosis	7	14	0.005	ARFGAP1, PRKCZ, CXCR4, CXCR1, CXCR2, DNM1, DNM2
KEGG_PATHWAY	hsa05100:Bacterial invasion of epithelial cells	7	14	8.68E-06	ACTB, SEPT3, ILK, CRK, DNM1, FN1, DNM2
KEGG_PATHWAY	hsa04510:Focal adhesion	6	12	0.010	ACTB, ILK, ZYX, CRK, VASP, FN1
KEGG_PATHWAY	hsa04611:Platelet activation	6	12	0.001	ACTB, PRKCZ, PRKACA, PRKG2, PRKG1, VASP
KEGG_PATHWAY	hsa05205:Proteoglycans in cancer	5	10	0.037	ACTB, EIF4B, PRKACA, STAT3, FN1
KEGG_PATHWAY	hsa04923:Regulation of lipolysis in adipocytes	3	6	0.049	PRKACA, PRKG2, PRKG1
KEGG_PATHWAY	hsa05110:Vibrio cholerae infection	3	6	0.045	ACTB, PRKACA, TJP2
KEGG_PATHWAY	hsa04961:Endocrine and other factor-regulated calcium reabsorption	3	6	0.033	PRKACA, DNM1, DNM2

Supplementary Table S6. The results of PANTHER pathway analysis of LASP-1 interactors.

Category	Term	Count	%	Genes
PANTHER pathway	Inflammation mediated by chemokine and cytokine signaling pathway (P00031)	11	7.60%	PRKCZ,PRKACA,ACTB,REL,STAT3,CXCR2, CXCR4, CXCR1,ACTC1,ACTA1,CXCR3
PANTHER pathway	CCKR signaling map (P06959)	9	6.30%	TCF4, PRKG1, PRKACA, PPP3CA, PRKACA, HSPB1, STAT3,CRK,DNM1
PANTHER pathway	Integrin signalling pathway (P00034)	8	5.60%	ILK, FN1, ACTB, CRK,ARFGAP1
PANTHER pathway	Angiogenesis (P00005)	6	4.20%	PRKCZ,PRKACA,HSPB1,STAT3, SH2D2A, CRK
PANTHER pathway	Endothelin signaling pathway (P00019)	6	4.20%	PRKG1,PRKCZ,PRKG2,PRKACA,PRKACA,PRKAR2B
PANTHER pathway	Gonadotropin-releasing hormone receptor pathway (P06664)	6	4.20%	PRKCZ,PRKACA,PPP3CA,STAT3,DYN1,NFYA
PANTHER pathway	Wnt signaling pathway (P00057)	6	4.20%	PRKCZ,PRKACA,PPP3CA,ACTB,ACTC1, ACTA1
PANTHER pathway	EGF receptor signaling pathway (P00018)	5	3.50%	PRKCZ,PRKACA,SFN, STAT3,SPRY2
PANTHER pathway	Cytoskeletal regulation by Rho GTPase (P00016)	4	2.80%	ACTB,ACTC1, ACTA1, VASP
PANTHER pathway	FGF signaling pathway (P00021)	4	2.80%	PRKCZ,PRKACA,SFN, SPRY2
PANTHER pathway	Huntington disease (P00029)	4	2.80%	DCTN1,ACTB,ACTC1,ACTA1
PANTHER pathway	VEGF signaling pathway (P00056)	4	2.80%	PRKCZ,PRKACA, HSPB1,SH2D2A
PANTHER pathway	Alzheimer disease-presenilin pathway (P00004)	3	2.10%	ACTB, ACTC1, ACTA1
PANTHER pathway	Cadherin signaling pathway (P00012)	3	2.10%	ACTB,ACTC1,ACTA1
PANTHER pathway	Interleukin signaling pathway (P00036)	3	2.10%	STAT3,CXCR2,CXCR1
PANTHER pathway	Nicotinic acetylcholine receptor signaling pathway (P00044)	3	2.10%	ACTB,ACTC1,ACTA1
PANTHER pathway	5HT1 type receptor mediated signaling pathway (P04373)	2	1.40%	PRKACA,PRKAR2B
PANTHER pathway	5HT2 type receptor mediated signaling pathway (P04374)	2	1.40%	PRKCZ, PRKACA
PANTHER pathway	Alzheimer disease-amyloid secretase pathway (P00003)	2	1.40%	PRKCZ, PRKACA
PANTHER pathway	Apoptosis signaling pathway (P00006)	2	1.40%	PRKACA, REL
PANTHER pathway	Beta1 adrenergic receptor signaling pathway (P04377)	2	1.40%	PRKACA,PRKAR2B
PANTHER pathway	Beta2 adrenergic receptor signaling pathway (P04378)	2	1.40%	PRKACA,PRKAR2B
PANTHER pathway	Dopamine receptor mediated signaling pathway (P05912)	2	1.40%	PRKACA,PRKAR2B
PANTHER pathway	Enkephalin release (P05913)	2	1.40%	PRKACA,PRKAR2B

Continued

Category	Term	Count	%	Genes
PANTHER pathway	GABA-B receptor II signaling (P05731)	2	1.40%	PRKACA, PRKAR2B
PANTHER pathway	Heterotrimeric G-protein signaling pathway-Gi alpha and Gs alpha mediated pathway (P00026)	2	1.40%	PRKACA,PRKAR2B
PANTHER pathway	Heterotrimeric G-protein signaling pathway-Gq alpha and Go alpha mediated pathway (P00027)	2	1.40%	PRKCZ, PRKACA
PANTHER pathway	Histamine H1 receptor mediated signaling pathway (P04385)	2	1.40%	PRKCZ,PRKACA
PANTHER pathway	Histamine H2 receptor mediated signaling pathway (P04386)	2	1.40%	PRKACA,PRKAR2B
PANTHER pathway	Metabotropic glutamate receptor group II pathway (P00040)	2	1.40%	PRKACA, PRKAR2B
PANTHER pathway	Metabotropic glutamate receptor group III pathway (P00039)	2	1.40%	PRKACA, PRKAR2B
PANTHER pathway	Muscarinic acetylcholine receptor 1 and 3 signaling pathway (P00042)	2	1.40%	PRKCZ, PRKACA
PANTHER pathway	Muscarinic acetylcholine receptor 2 and 4 signaling pathway (P00043)	2	1.40%	PRKACA, PRKAR2B
PANTHER pathway	Oxytocin receptor mediated signaling pathway (P04391)	2	1.40%	PRKCZ,PRKACA
PANTHER pathway	p53 pathway (P00059)	2	1.40%	SFN,SUMO2
PANTHER pathway	Parkinson disease (P00049)	2	1.40%	PSA3,SFN
PANTHER pathway	PDGF signaling pathway (P00047)	2	1.40%	PRKACA,STAT3
PANTHER pathway	Thyrotropin-releasing hormone receptor signaling pathway (P04394)	2	1.40%	PRKCZ,PRKACA
PANTHER pathway	Alpha adrenergic receptor signaling pathway (P00002)	1	0.70%	PRKACA
PANTHER pathway	Angiotensin II-stimulated signaling through G proteins and beta-arrestin (P05911)	1	0.70%	PRKACA
PANTHER pathway	Axon guidance mediated by netrin (P00009)	1	0.70%	CXCR4
PANTHER pathway	Axon guidance mediated by Slit/Robo (P00008)	1	0.70%	VASP
PANTHER pathway	B cell activation (P00010)	1	0.70%	PPP3CA
PANTHER pathway	Hedgehog signaling pathway (P00025)	1	0.70%	PRKAR2B
PANTHER pathway	Heterotrimeric G-protein signaling pathway-rod outer segment phototransduction (P00028)	1	0.70%	PRKACA
PANTHER pathway	JAK/STAT signaling pathway (P00038)	1	0.70%	STAT3
PANTHER pathway	Metabotropic glutamate receptor group I pathway (P00041)	1	0.70%	PRKACA
PANTHER pathway	Nicotine pharmacodynamics pathway (P06587)	1	0.70%	PRKACA
PANTHER pathway	p38 MAPK pathway (P05918)	1	0.70%	HSPB1
PANTHER pathway	Ras Pathway (P04393)	1	0.70%	STAT3
PANTHER pathway	T cell activation (P00053)	1	0.70%	PPP3CA
PANTHER pathway	Toll receptor signaling pathway (P00054)	1	0.70%	REL
PANTHER pathway	Transcription regulation by bZIP transcription factor (P00055)	1	0.70%	PRKAR2B

Supplementary Table S7. The interaction information of LASP-1 interactors from STRING database

#node1	#node2	node1_string_ internal_id	node2_string_ internal_id	experimentally_ determined_interaction	database_ annotated	automated_ textmining	combined_score
CXCR4	UBC	1859216	1852861	0.999	0	0	0.999
POT1	TINF2	1853560	1845941	0.99	0.9	0.87	0.999
ZYX	VASP	1850641	1843785	0.914	0.9	0.983	0.999
ACTA1	UBC	1854428	1852861	0.998	0	0.243	0.999
POT1	TERF1	1853560	1846506	0.693	0.9	0.973	0.999
PRKACA	PRKAR2B	1849201	1845791	0.959	0.9	0.932	0.999
ACD	POT1	1858140	1853560	0.997	0.9	0.949	0.999
ACTB	UBC	1853523	1852861	0.998	0	0.511	0.999
SUMO2	UBC	1860567	1852861	0.997	0	0.594	0.999
ACD	TINF2	1858140	1845941	0.973	0.9	0.948	0.999
TERF1	TINF2	1846506	1845941	0.999	0.9	0.973	0.999
UBC	PSMA3	1852861	1842599	0.987	0.9	0	0.998
UBC	HSPB1	1852861	1843915	0.98	0.9	0.128	0.998
UBC	STAT3	1852861	1845584	0.997	0	0.165	0.998
LPP	VASP	1850011	1843785	0.576	0.9	0.936	0.997
SPRY2	UBC	1856611	1852861	0.961	0.9	0	0.996
UBC	DDB1	1852861	1848334	0.996	0	0	0.996
UBC	ACTC1	1852861	1847228	0.993	0	0.201	0.994
UBC	TERF1	1852861	1846506	0.994	0	0.043	0.994
UBC	VIM	1852861	1842995	0.993	0	0.13	0.994
PRKG1	VASP	1856026	1843785	0.576	0.8	0.943	0.994
ACD	TERF1	1858140	1846506	0.796	0.9	0.69	0.993
UBC	SFN	1852861	1852398	0.991	0	0.107	0.992
PRKG2	VASP	1845539	1843785	0	0.8	0.897	0.978
PRKCZ	UBC	1856893	1852861	0.731	0.9	0.082	0.973
CXCR4	STAT3	1859216	1845584	0.57	0.9	0.191	0.962
DCTN1	UBC	1854219	1852861	0.957	0	0	0.958
UBC	HAX1	1852861	1851101	0.958	0	0	0.958
UBC	RDH12	1852861	1845950	0.957	0	0	0.957
CXCR2	CXCR1	1850165	1847603	0.576	0.9	0.98	0.956
TCF4	UBC	1853052	1852861	0.951	0	0.109	0.955
ZYX	ILK	1850641	1848060	0	0.9	0.532	0.951
FBF1	SCLT1	1850631	1846708	0	0.9	0.473	0.945
UBC	S100A11	1852861	1846206	0.944	0	0	0.944
PPP3CA	UBC	1858236	1852861	0.937	0	0.116	0.942
ACTA1	VASP	1854428	1843785	0.576	0	0.861	0.938
PPP3CA	DNM1	1858236	1855784	0.101	0.9	0.287	0.937
**Continued**							

#node1	#node2	node1_string_ internal_id	node2_string_ internal_id	experimentally_ determined_interaction	database_ annotated	automated_ textmining	combined_score
UBC	ILK	1852861	1848060	0.928	0	0.092	0.932
UBC	FANCC	1852861	1847153	0.296	0.9	0	0.926
FHL3	UBC	1855805	1852861	0.925	0	0	0.925
UBC	PRKACA	1852861	1849201	0.911	0	0.142	0.924
PRKACA	VASP	1849201	1843785	0.576	0.8	0.176	0.924
ACTA1	ZYX	1854428	1850641	0	0.9	0.262	0.923
MAP4	UBC	1853972	1852861	0.922	0	0.046	0.923
AHNAK	UBC	1856799	1852861	0.923	0	0	0.923
DNM1	DNM2	1855784	1853228	0.193	0.9	0.942	0.917
CXCR4	CXCR2	1859216	1850165	0	0.9	0.889	0.916
REL	STAT3	1847519	1845584	0	0	0.916	0.916
ACTA1	HSPB1	1854428	1843915	0.077	0.9	0.129	0.912
HSPB1	PSMA3	1843915	1842599	0	0.9	0.159	0.912
CXCR3	CXCR2	1855969	1850165	0	0.9	0.829	0.911
PRKACA	CDK7	1849201	1844436	0	0.9	0.384	0.911
CXCR3	CXCR1	1855969	1847603	0	0.9	0.68	0.909
FN1	CRK	1853098	1848191	0	0.9	0.121	0.908
UBC	CD81	1852861	1845382	0.907	0	0.056	0.908
DNM1	CXCR2	1855784	1850165	0	0.9	0.102	0.906
CXCR4	CXCR3	1859216	1855969	0	0.9	0.506	0.906
CXCR4	CXCR1	1859216	1847603	0	0.9	0.348	0.905
CXCR2	VASP	1850165	1843785	0	0.9	0.072	0.903
UBC	LGALS1	1852861	1842521	0.897	0	0.092	0.903
CXCR3	DNM1	1855969	1855784	0	0.9	0.061	0.902
CXCR4	DNM1	1859216	1855784	0	0.9	0.066	0.902
SFN	CDK7	1852398	1844436	0	0.9	0.067	0.902
FN1	PRKACA	1853098	1849201	0	0.9	0.045	0.9
DNM1	CXCR1	1855784	1847603	0	0.9	0.047	0.9
TAS2R41	CXCR2	1859127	1850165	0	0.9	0	0.9
TAS2R41	CXCR3	1859127	1855969	0	0.9	0	0.9
TAS2R41	CXCR1	1859127	1847603	0	0.9	0	0.9
CXCR4	TAS2R41	1859216	1859127	0	0.9	0	0.9
STAT3	HSPB1	1845584	1843915	0.073	0	0.889	0.893
DAZAP2	UBC	1862477	1852861	0.891	0	0	0.891
ACTB	DNM2	1853523	1853228	0.631	0	0.699	0.888
UBC	TRIP13	1852861	1842297	0.877	0	0.106	0.886
UBC	ARFGAP1	1852861	1849722	0.882	0	0	0.882
UBC	NXF1	1852861	1847469	0.874	0	0.041	0.875
UBC	HSPE1	1852861	1843372	0.855	0	0.163	0.874
NXF1	FYTTD1	1847469	1843600	0.804	0	0.368	0.871
DNM2	UBC	1853228	1852861	0.845	0	0.088	0.861

Continued

#node1	#node2	node1_string_ internal_id	node2_string_ internal_id	experimentally_ determined_interaction	database_ annotated	automated_ textmining	combined_score
PLSCR1	UBC	1852944	1852861	0.854	0	0	0.854
TCF4	STAT3	1853052	1845584	0	0	0.846	0.846
ACTB	VASP	1853523	1843785	0	0.8	0.217	0.836
UBC	PRKAR2B	1852861	1845791	0.809	0	0.139	0.834
DNM1	UBC	1855784	1852861	0.813	0	0.066	0.828
ACTB	ZYX	1853523	1850641	0	0.8	0.152	0.823
PRKG1	HSPB1	1856026	1843915	0	0	0.815	0.815
UBC	EIF4B	1852861	1845052	0.811	0	0	0.812
FN1	UBC	1853098	1852861	0.781	0	0.168	0.81
DCTN1	ACTB	1854219	1853523	0.3	0	0.726	0.807
TJP2	ACTB	1862087	1853523	0	0.8	0.067	0.805
SUMO2	ACTB	1860567	1853523	0.747	0	0.211	0.798
UBC	VASP	1852861	1843785	0.795	0	0.052	0.797
PRKAR2B	PRKG2	1845791	1845539	0.45	0	0.754	0.774
PRKG1	PRKAR2B	1856026	1845791	0.45	0	0.75	0.774
DNM2	ACTC1	1853228	1847228	0.19	0	0.72	0.771
DCTN1	ACTC1	1854219	1847228	0.3	0	0.674	0.77
ACTA1	DCTN1	1854428	1854219	0.3	0	0.674	0.77
ACTB	ARFGAP1	1853523	1849722	0.261	0	0.696	0.766
ACTA1	ARFGAP1	1854428	1849722	0.261	0	0.696	0.766
ARFGAP1	ACTC1	1849722	1847228	0.261	0	0.696	0.766
ACTA1	DNM2	1854428	1853228	0.19	0	0.686	0.743
PALLD	UBC	1861658	1852861	0.721	0	0.105	0.74
TJP2	UBC	1862087	1852861	0.721	0	0.092	0.736
LPP	CRK	1850011	1848191	0	0	0.651	0.732
GOLGA2	UBC	1861205	1852861	0.73	0	0	0.73
UBC	FXR2	1852861	1843993	0.721	0	0	0.721
UBC	ZYX	1852861	1850641	0.721	0	0	0.721
DNM1	ACTB	1855784	1853523	0.19	0	0.647	0.711
CRK	HSPB1	1848191	1843915	0.644	0	0.214	0.708
DNM1	ACTA1	1855784	1854428	0.19	0	0.633	0.699
UBC	KBTBD10	1852861	1846919	0.686	0	0.079	0.699
ACTA1	VIM	1854428	1842995	0.57	0	0.328	0.699
DNM1	ACTC1	1855784	1847228	0.19	0	0.633	0.699
3-Sep	UBC	1858396	1852861	0.666	0	0.092	0.684
PPP3CA	PRKCZ	1858236	1856893	0.576	0	0.271	0.677
ZYX	VIM	1850641	1842995	0.644	0	0.112	0.67
FN1	ZYX	1853098	1850641	0.59	0	0.202	0.659
DAZAP2	RBPMS	1862477	1852294	0.633	0	0.109	0.659
TJP2	ZYX	1862087	1850641	0.57	0	0.23	0.655
DDB1	NXF1	1848334	1847469	0.644	0	0	0.644

Continued

#node1	#node2	node1_string_ internal_id	node2_string_ internal_id	experimentally_ determined_interaction	database_ annotated	automated_ textmining	combined_score
PALLD	HSPB1	1861658	1843915	0.644	0	0	0.644
DNM2	CRK	1853228	1848191	0.288	0	0.477	0.64
SUMO2	ATXN1	1860567	1843741	0.573	0	0.163	0.627
CEP170	UBC	1854392	1852861	0.621	0	0	0.621
UBC	FYTTD1	1852861	1843600	0.62	0	0	0.62
LZTS2	UBC	1855200	1852861	0.62	0	0	0.62
ZDHHC17	SPRY2	1860403	1856611	0.614	0	0	0.614
ACTB	S100A11	1853523	1846206	0.576	0	0.128	0.614
TJP2	LPP	1862087	1850011	0.57	0	0.125	0.608
ACTC1	HSPB1	1847228	1843915	0.591	0	0.081	0.608
DAZAP2	RHOXF2	1862477	1855471	0.607	0	0	0.607
HSPB1	HSPE1	1843915	1843372	0	0	0.606	0.606
FHL3	ACTB	1855805	1853523	0.576	0	0.105	0.604
UBC	ATXN1	1852861	1843741	0.564	0	0.112	0.596
PRKCZ	STAT3	1856893	1845584	0.576	0	0.071	0.589
3-Sep	PRKG1	1858396	1856026	0.576	0	0.068	0.587
ACTB	ACTC1	1853523	1847228	0.576	0	0.774	0.579
UBC	TINF2	1852861	1845941	0.576	0	0	0.576
DCTN1	VIM	1854219	1842995	0.576	0	0	0.576
PRKACA	VIM	1849201	1842995	0.576	0	0	0.576
SUMO2	AHNAK	1860567	1856799	0.562	0	0	0.562
ZYX	CRK	1850641	1848191	0	0	0.505	0.557
PLSCR1	ZNF764	1852944	1844160	0.506	0	0.104	0.556
NXF1	FXR2	1847469	1843993	0	0	0.554	0.554
RHOXF2	LZTS2	1855471	1855200	0.553	0	0	0.553
PPP3CA	PRKACA	1858236	1849201	0.101	0	0.203	0.54
ACTB	VIM	1853523	1842995	0.151	0	0.477	0.537
PPP3CA	PRKG2	1858236	1845539	0.101	0	0.186	0.53
PPP3CA	PRKG1	1858236	1856026	0.101	0	0.186	0.53
ACTB	CRK	1853523	1848191	0.517	0	0.058	0.525
UBC	CRK	1852861	1848191	0.498	0	0.068	0.513
DNM2	ZYX	1853228	1850641	0	0	0.511	0.511
DNM2	VASP	1853228	1843785	0	0	0.507	0.507
TJP2	PRKCZ	1862087	1856893	0	0	0.504	0.504
PPP3CA	PRKAR2B	1858236	1845791	0.1	0	0.377	0.5
NFYA	UBC	1852963	1852861	0.5	0	0	0.5
RBPMS	ATXN1	1852294	1843741	0.294	0	0.313	0.494
SUMO2	STAT3	1860567	1845584	0.308	0	0.289	0.487
ACTB	RBPMS	1853523	1852294	0	0	0.481	0.481
ZDHHC17	ARFGAP1	1860403	1849722	0.466	0	0.064	0.478
ZDHHC17	UBC	1860403	1852861	0.447	0	0.074	0.466

Continued

#node1	#node2	node1_string_ internal_id	node2_string_ internal_id	experimentally_ determined_interaction	database_ annotated	automated_ textmining	combined_score
SFN	PRKACA	1852398	1849201	0.345	0	0.2	0.453
PRKG1	SFN	1856026	1852398	0.345	0	0.2	0.453
SFN	PRKG2	1852398	1845539	0.345	0	0.2	0.453
CRK	ILK	1848191	1848060	0	0	0.449	0.449
HSPB1	VIM	1843915	1842995	0.105	0	0.407	0.446
VIM	LGALS1	1842995	1842521	0	0	0.396	0.44
LZTS2	RBPMS	1855200	1852294	0.435	0	0	0.435
PRKAR2B	CDK7	1845791	1844436	0	0.36	0.15	0.433
FN1	VIM	1853098	1842995	0.081	0	0.398	0.423
SUMO2	NXF1	1860567	1847469	0.397	0	0.081	0.422
CXCR3	STAT3	1855969	1845584	0	0	0.418	0.418
CRK	ATXN1	1848191	1843741	0.416	0	0	0.416
FN1	RBPMS	1853098	1852294	0	0	0.412	0.412
PLSCR1	PRKACA	1852944	1849201	0	0	0.071	0.407
ILK	STAT3	1848060	1845584	0	0	0.406	0.406

Supplementary Table S8. The relative expression levels of LASP-1 and its interactors in microarray of HBV-related HCC tissues.

Transcript Cluster ID	Gene Symbol	Non-HCC Bi-weight Avg Signal (log2)	HBV-HCC Bi-weight Avg Signal (log2)	Fold Change (linear) (Non-HCC vs. HBV-HCC)
204617_s_at	ACD	4.38	5.18	-1.74
203872_at	ACTA1	3.37	3.53	-1.12
200801_x_at	ACTB	12.26	12.79	-1.44
213867_x_at	ACTB	12.03	12.6	-1.49
205132_at	ACTC1	3.68	4.04	-1.28
211986_at	AHNAK	7.45	8.57	-2.17
217888_s_at	ARFGAP1	4.45	5.77	-2.5
205507_at	ARHGEF15	3.51	3.82	-1.24
217348_x_at	ARHGEF15	3.69	4.09	-1.31
203232_s_at	ATXN1	6.51	7.23	-1.66
203231_s_at	ATXN1	5	5.33	-1.26
—	C11orf65	—	—	—
—	CCDC8	—	—	—
200675_at	CD81	9.94	9.38	1.48
211297_s_at	CDK7	4.45	6.17	-3.3
207719_x_at	CEP170	6.13	7.25	-2.17
212746_s_at	CEP170	4.11	5.24	-2.18
202226_s_at	CRK	5.7	5.96	-1.2
202225_at	CRK	7.87	7.95	-1.06
202224_at	CRK	6.42	6.57	-1.1
207094_at	CXCR1	3.92	4.16	-1.19
—	CXCR2	—	—	—
207681_at	CXCR3	3.53	3.98	-1.36
217119_s_at	CXCR3	3.66	3.81	-1.11
217028_at	CXCR4	6.83	7.32	-1.4
209201_x_at	CXCR4	4.91	5.3	-1.31
211919_s_at	CXCR4	4.93	5.26	-1.26
212595_s_at	DAZAP2	4.82	6.47	-3.13
200794_x_at	DAZAP2	9.16	9.85	-1.61
214334_x_at	DAZAP2	8.68	9.45	-1.71
201082_s_at	DCTN1	5.2	6.35	-2.22
211780_x_at	DCTN1	5.65	6.69	-2.05
204296_at	DCTN1	3.28	3.58	-1.23
208619_at	DDB1	6.98	8.2	-2.33
219166_at	DNAAF2	4.28	5.66	-2.6
215116_s_at	DNM1	3.9	4.38	-1.4
217341_at	DNM1	3.92	4.21	-1.22

Continued

Transcript Cluster ID	Gene Symbol	Non-HCC Bi-weight Avg Signal (log2)	HBV-HCC Bi-weight Avg Signal (log2)	Fold Change (linear) (Non-HCC vs. HBV-HCC)
202253_s_at	DNM2	5.66	5.24	1.34
216024_at	DNM2	3.14	3.5	-1.29
211937_at	EIF4B	8.18	8.95	-1.7
211938_at	EIF4B	9.32	9.61	-1.23
205189_s_at	FANCC	6.21	4.98	2.35
—	FBF1	—	—	—
218818_at	FHL3	3.58	4.18	-1.52
214702_at	FN1	4.26	3.95	1.23
214701_s_at	FN1	4.86	5.02	-1.12
216442_x_at	FN1	12.42	12.58	-1.12
35265_at	FXR2	4.72	4.95	-1.17
203172_at	FXR2	4.66	4.84	-1.14
—	FYTTD1	—	—	—
204384_at	GOLGA2	4.01	4.66	-1.57
211059_s_at	GOLGA2	4.84	5.29	-1.37
35436_at	GOLGA2	6.67	7.15	-1.4
201145_at	HAX1	7.46	8.95	-2.82
201841_s_at	HSPB1	8.53	10.77	-4.71
205133_s_at	HSPE1	9.41	10.48	-2.09
201234_at	ILK	6.66	7.7	-2.06
—	KRTAP4-2	—	—	—
219106_s_at	KLHL41	3.03	3.29	-1.2
200618_at	LASP1	8.42	9.66	-2.37
201105_at	LGALS1	8.52	9.24	-1.65
202821_s_at	LPP	3.83	4.63	-1.74
202822_at	LPP	7.2	8.1	-1.88
—	LZTS2	—	—	—
200836_s_at	MAP4	4.53	6.59	-4.17
212566_at	MAP4	6.25	7.69	-2.72
212567_s_at	MAP4	4.71	5.84	-2.19
243_g_at	MAP4	6.09	7.64	-2.93
200835_s_at	MAP4	3.92	4.82	-1.86
33850_at	MAP4	4.6	5.55	-1.93
205375_at	MDFI	3.4	3.61	-1.16
204108_at	NFYA	4.06	5.56	-2.82
204107_at	NFYA	3.41	4.13	-1.65
204109_s_at	NFYA	3.94	4.65	-1.63
215720_s_at	NFYA	3.55	3.88	-1.26
208922_s_at	NXF1	7.66	7.57	1.07
208520_at	OR10H3	3.42	3.66	-1.18
200897_s_at	PALLD	6.96	8.67	-3.28
**Continued**				

Transcript Cluster ID	Gene Symbol	Non-HCC Bi-weight Avg Signal (log2)	HBV-HCC Bi-weight Avg Signal (log2)	Fold Change (linear) (Non-HCC vs. HBV-HCC)
200906_s_at	PALLD	4.43	5.95	-2.85
200907_s_at	PALLD	5.58	7.36	-3.44
202430_s_at	PLSCR1	5.93	6.68	-1.68
202446_s_at	PLSCR1	7.21	7.63	-1.34
204353_s_at	POT1	3.83	4.93	-2.15
204354_at	POT1	5.11	6.3	-2.27
202425_x_at	PPP3CA	5.49	5.97	-1.4
202429_s_at	PPP3CA	6.33	6.84	-1.42
202457_s_at	PPP3CA	5.74	6.26	-1.44
216234_s_at	PRKACA	3.18	3.42	-1.17
202801_at	PRKACA	5.84	6.14	-1.24
—	PRKAR2B	—	—	—
202178_at	PRKCZ	5.79	6.15	-1.28
207119_at	PRKG1	3.28	3.51	-1.18
211380_s_at	PRKG1	3.31	3.61	-1.23
207505_at	PRKG2	3.31	3.47	-1.12
201532_at	PSMA3	8.21	9.42	-2.31
209487_at	RBPMS	6.22	7.39	-2.24
207836_s_at	RBPMS	4.58	5.11	-1.45
209488_s_at	RBPMS	6.59	7.45	-1.81
207837_at	RBPMS	4.21	3.93	1.22
—	RDH12	—	—	—
206035_at	REL	3.19	3.47	-1.21
—	RHOXF2	—	—	—
200660_at	S100A11	6.11	7.46	-2.56
—	SCLT1	—	—	—
—	SEPT3	—	—	—
33322_i_at	SFN	5.36	7.53	-4.5
33323_r_at	SFN	4.11	7.1	-7.94
209260_at	SFN	3.26	3.97	-1.63
207351_s_at	SH2D2A	3.84	4.13	-1.22
221020_s_at	SLC25A32	6.12	7.06	-1.92
32091_at	SLC25A44	7.68	8.74	-2.09
212683_at	SLC25A44	5.43	5.99	-1.48
212438_at	SNRNP27	5.19	6.29	-2.14
212440_at	SNRNP27	6.07	7.37	-2.47
204011_at	SPRY2	6.1	5.73	1.29
208992_s_at	STAT3	7.58	8.09	-1.42
208991_at	STAT3	8.75	8.95	-1.15
208739_x_at	SUMO2	8.24	9.58	-2.53
213881_x_at	SUMO2	9.81	11.04	-2.35

Continued

Transcript Cluster ID	Gene Symbol	Non-HCC Bi-weight Avg Signal (log2)	HBV-HCC Bi-weight Avg Signal (log2)	Fold Change (linear) (Non-HCC vs. HBV-HCC)
208738_x_at	SUMO2	8.99	9.99	-2
211444_at	SUMO2	3.36	3.84	-1.39
—	TAS2R41	—	—	—
203753_at	TCF4	5.87	6.6	-1.66
212385_at	TCF4	3.53	4.04	-1.42
212387_at	TCF4	4.43	4.99	-1.48
212382_at	TCF4	5.29	5.69	-1.31
213891_s_at	TCF4	6.88	7.23	-1.27
212386_at	TCF4	6.75	7.07	-1.25
222146_s_at	TCF4	5.76	5.6	1.12
203448_s_at	TERF1	4.95	6.35	-2.63
203449_s_at	TERF1	4.87	6.4	-2.89
219292_at	THAP1	3.73	4.57	-1.79
—	THAP8	—	—	—
220052_s_at	TINF2	4.36	5.11	-1.67
—	TJP2	—	—	—
210541_s_at	TRIM27	5.49	6.79	-2.46
212116_at	TRIM27	5.82	7.27	-2.74
212118_at	TRIM27	6.38	7.46	-2.11
204033_at	TRIP13	3.55	4.98	-2.71
208980_s_at	UBC	10.73	11.42	-1.62
211296_x_at	UBC	12.25	12.73	-1.39
202205_at	VASP	4.78	6.28	-2.84
201426_s_at	VIM	8.72	9.48	-1.7
—	ZBTB9	—	—	—
205308_at	ZC2HC1A	4.62	5.07	-1.37
212982_at	ZDHHC17	6.02	6.75	-1.67
217486_s_at	ZDHHC17	3.45	3.78	-1.26
222120_at	ZNF764	4.21	4.54	-1.26
57516_at	ZNF764	3.38	4.26	-1.84
200808_s_at	ZYX	5.48	7	-2.87
215706_x_at	ZYX	5.49	7.27	-3.44

Note: — represent the expression of target gene was not found in the microarray of GSE14520.

Supplementary Table S9. The co-expression pairs of LASP-1 and its interactors assessed by DeGNServer online platform.

Protein 1	Protein 2	Pearson's correlation coefficients
DAZAP2	TERF1	4.59762
HSPB1	ZYX	3.61114
S100A11	SFN	3.59299
PSMA3	SNRNP27	3.58114
ILK	ZYX	3.53637
HAX1	SLC25A44	3.34156
RBPMS	VASP	3.26024
HSPE1	NFYA	3.11182
ILK	MAP4	3.08684
DNAAF2	SNRNP27	3.06118
DAZAP2	POT1	2.94824
MAP4	PALLD	2.93648
DCTN1	ILK	2.93029
CDK7	SUMO2	2.92253
DCTN1	DDB1	2.87983
ARFGAP1	TRIP13	2.8645
CDK7	HSPE1	2.85761
NFYA	TRIM27	2.77077
DNAAF2	PSMA3	2.76802
PALLD	VASP	2.74322
NFYA	SUMO2	2.73167
MAP4	ZYX	2.70627
MAP4	RBPMS	2.66378
S100A11	SLC25A44	2.65499
CDK7	CEP170	2.65095
S100A11	VASP	2.64176
POT1	RBPMS	2.62291
DDB1	ILK	2.44568
FANCC	ILK	2.44406
POT1	SNRNP27	2.44311
CDK7	FANCC	2.42404
HAX1	TRIM27	2.41452
DDB1	DNAAF2	2.3987
SUMO2	TRIP13	2.37298
CDK7	DNAAF2	2.3498
LASP1	SUMO2	2.34891
SFN	TRIP13	2.34472
ARFGAP1	PSMA3	2.30608
**Continued**		

Protein 1	Protein 2	Pearson’s correlation coefficients
LASP1	TRIP13	2.28163
DNAAF2	TRIM27	2.21866
DCTN1	ZYX	2.20472
PALLD	RBPMS	2.13799
AHNAK	ZYX	2.12963
HSPE1	LASP1	2.12387
SNRNP27	SUMO2	2.07341
PALLD	SLC25A44	2.01285
RBPMS	S100A11	2.00814
CEP170	TRIP13	1.97586
LASP1	NFYA	1.96001
AHNAK	TRIP13	1.95628
ILK	RBPMS	1.89296
AHNAK	HSPB1	1.86337
ARFGAP1	SNRNP27	1.83766
HSPB1	LASP1	1.83733
CEP170	SUMO2	1.83234
DCTN1	HSPB1	1.82596
SNRNP27	TERF1	1.79568
PALLD	S100A11	1.77726
PSMA3	SUMO2	1.73649
HSPB1	MAP4	1.72986
CEP170	NFYA	1.72581
TERF1	VASP	1.69169
DDB1	SNRNP27	1.66298
LASP1	ZYX	1.65837
SLC25A44	VASP	1.64974
PALLD	TERF1	1.64972
CDK7	PSMA3	1.64099
POT1	S100A11	1.63918
AHNAK	ILK	1.63403
AHNAK	MAP4	1.6302
DNAAF2	S100A11	1.61729
DCTN1	HAX1	1.56169
HSPB1	SFN	1.53964
HSPE1	SUMO2	1.53938
DNAAF2	TERF1	1.5024
CDK7	NFYA	1.49688
CDK7	TRIP13	1.45701
HSPB1	ILK	1.43439
MAP4	S100A11	1.42058
HAX1	PSMA3	1.3999
**Continued**		

Protein 1	Protein 2	Pearson’s correlation coefficients
DNAAF2	POT1	1.38331
HSPE1	TRIP13	1.36258
DCTN1	SNRNP27	1.36201
HAX1	SNRNP27	1.35591
DAZAP2	FANCC	1.35494
CDK7	TRIM27	1.35265
PALLD	SFN	1.35109
SLC25A44	SUMO2	1.33204
DNAAF2	NFYA	1.2664
CEP170	VASP	1.24996
DDB1	TRIM27	1.23325
MAP4	VASP	1.19253
CEP170	S100A11	1.15511
LASP1	S100A11	1.1537
CDK7	LASP1	1.14403
S100A11	ZYX	1.13795
AHNAK	VASP	1.12853
HSPB1	S100A11	1.12318
DNAAF2	HSPE1	1.1165
ARFGAP1	DAZAP2	1.11593
DDB1	ZYX	1.06474
LASP1	VASP	1.06119
DAZAP2	ILK	1.05748
PSMA3	TRIM27	1.05308
ILK	PALLD	1.05285
FANCC	HAX1	1.05054
SUMO2	VASP	1.0365
DCTN1	NFYA	1.02377
ARFGAP1	DCTN1	1.01766
CDK7	DDB1	1.0115
S100A11	TRIP13	1.00588
FANCC	TERF1	1.00133
FANCC	MAP4	0.997376
CDK7	HAX1	0.990429
DDB1	FANCC	0.984017
NFYA	PALLD	0.955374
DDB1	PSMA3	0.936559
AHNAK	CEP170	0.921959
HSPE1	SNRNP27	0.886695
DDB1	SUMO2	0.873422
DNAAF2	ILK	0.859829
HAX1	SFN	0.8589
**Continued**		

Protein 1	Protein 2	Pearson's correlation coefficients
AHNAK	DCTN1	0.858063
SLC25A44	TRIP13	0.848413
TRIM27	VASP	0.813746
DAZAP2	ZYX	0.813605
DCTN1	SUMO2	0.800841

Supplementary Table S10. The results of predicated transcription factors of LASP-1 interactors by Enrichr online tool.

Term	Species	P-value	Z-score	Genes
CRTC3	human	0.019829	-1.60043	DAZAP2;PALLD;ILK;MAP4
ELK1	human	0.018759	-1.57145	PALLD;DCTN1;SUMO2;POT1;HSPE1
ETS2	human	0.048867	-1.30057	LASP1;NFYA;ILK;SFN
FOXC1	human	0.014688	-1.68007	VASP;DAZAP2;RBPMS;NFYA;DCTN1;HSPB1;ILK;FANCC;TERF1;HSPE1;HAX1;PALLD;SUMO2;ZYX;SNRNP27;POT1;CEP170;TRIM27;MAP4;S100A11
FOXJ1	human	0.017483	-1.5569	VASP;HAX1;HSPB1;TERF1;S100A11
HNF4A	human	0.005275	-1.60113	DAZAP2;PALLD;POT1;ILK;FANCC;TRIM27;MAP4
HOXD9	human	0.02035	-1.49373	PSMA3;HAX1;AHNAK;PALLD;HSPB1
JDP2	human	0.003819	-1.68712	PALLD;DCTN1;SUMO2;POT1;SNRNP27;HSPB1
JUN	human	0.009252	-1.51883	VASP;LASP1;AHNAK;PALLD;ZYX;HSPB1;FANCC;MAP4
LEF1	human	0.041972	-1.11731	LASP1;HAX1;AHNAK;NFYA;DCTN1;SUMO2;SNRNP27;ILK;HSPB1
NKX3-1	human	0.002631	-2.01339	VASP;PALLD;HSPB1
NR2F1	human	0.024291	-1.57178	DDB1;CDK7;ZYX;SNRNP27
NR5A2	human	0.003837	-1.48713	VASP;DDB1;DAZAP2;PSMA3;PALLD;DCTN1;ILK;CEP170;MAP4;ARFGAP1
PPARG	human	0.02624	-1.31809	DAZAP2;HAX1;PALLD;POT1;SFN;TERF1;HSPE1;ARFGAP1
PURA	human	0.014387	-1.73892	LASP1;DCTN1;TRIP13
RBPJ	human	0.019944	-1.5481	VASP;PSMA3;CDK7;SNRNP27;ILK
RORB	human	0.021614	-1.56109	DDB1;CDK7;ZYX;SNRNP27
TBP	human	0.012114	-1.47645	CDK7;DCTN1;ZYX;POT1;TRIP13;TERF1;S100A11
TFAP2C	human	0.003782	-1.6504	VASP;DAZAP2;AHNAK;DCTN1;TRIP13;TERF1
TFAP2D	human	0.047006	-1.38031	DAZAP2;CDK7;TRIM27;S100A11
ZBTB16	human	0.004994	-1.6557	NFYA;PALLD;DCTN1;CEP170;TRIM27;TERF1
ZNF354C	human	0.01511	-1.81506	CDK7;LASP1;DCTN1;ILK;TRIP13

Supplementary Table S11. The interaction information of predicated transcription factors from STRING database.

#node1	#node2	node1_string_internal_id	node2_string_internal_id	experimentally_determined_interaction	database_annotated	automated_textmining	combined_score
JUN	JDP2	1855436	1845956	0.874	0.9	0.968	0.999
JUN	ETS2	1855436	1853968	0.777	0.9	0.972	0.999
JUN	TBP	1855436	1843249	0.935	0	0.758	0.983
HNF4A	LEF1	1849587	1845704	0.091	0	0.962	0.964
NR2F1	HNF4A	1850774	1849587	0.579	0.9	0.619	0.96
JUN	ELK1	1855436	1843855	0.204	0.9	0.459	0.953
HNF4A	TBP	1849587	1843249	0.57	0	0.843	0.929
JUN	HNF4A	1855436	1849587	0	0	0.927	0.927
HNF4A	PPARG	1849587	1847090	0.075	0.9	0.627	0.922
RORB	NR2F1	1856572	1850774	0	0.9	0.513	0.915
RORB	PPARG	1856572	1847090	0	0.9	0.349	0.91
JUN	LEF1	1855436	1845704	0	0	0.908	0.908
NR2F1	PPARG	1850774	1847090	0	0.9	0.301	0.907
RORB	HNF4A	1856572	1849587	0	0.9	0.204	0.905
FOXJ1	LEF1	1850591	1845704	0.097	0	0.87	0.878
ETS2	TBP	1853968	1843249	0	0.8	0.306	0.855
ELK1	TBP	1843855	1843249	0	0.8	0.053	0.802
JUN	NR5A2	1855436	1854575	0	0	0.707	0.707
JUN	PPARG	1855436	1847090	0	0	0.633	0.633
JUN	RBPJ	1855436	1852908	0	0	0.407	0.407
HNF4A	ELK1	1849587	1843855	0	0	0.4	0.4

Supplementary Table S12. The relative expression and fold change of predicted transcription factors of LASP-1 interactors in microarray of HBV-related HCC tissues.

**Transcript Cluster ID**	**Gene Symbol**	**Non-HCC Bi-weight Avg Signal (log2)**	**HBV-HCC Bi-weight Avg Signal (log2)**	**Fold Change (linear) (Non-HCC vs. HBV-HCC)**
218648_at	CRTC3	6.7	7.73	-2.04
203617_x_at	ELK1	5.69	6.1	-1.32
210850_s_at	ELK1	3.41	3.75	-1.27
210376_x_at	ELK1	4.2	4.31	-1.08
201329_s_at	ETS2	6.97	6.52	1.36
201328_at	ETS2	8.8	8.22	1.5
213260_at	FOXC1	3.89	4.52	-1.55
205906_at	FOXJ1	3.79	4.09	-1.23
208429_x_at	HNF4A	4.17	4.65	-1.39
216889_s_at	HNF4A	3.78	4.04	-1.2
214832_at	HNF4A	5.01	5.16	-1.1
214851_at	HNF4A	3.7	3.78	-1.05
205604_at	HOXD9	3.5	3.89	-1.3
—	JDP2	—	—	—
201464_x_at	JUN	8.22	7.86	1.28
201465_s_at	JUN	6.18	5.76	1.33
221558_s_at	LEF1	3.43	4.65	-2.33
210948_s_at	LEF1	3.23	3.61	-1.3
221557_s_at	LEF1	3.44	3.74	-1.24
211497_x_at	NKX3-1	3.66	3.99	-1.26
211498_s_at	NKX3-1	3.63	3.95	-1.25
209706_at	NKX3-1	3.77	3.82	-1.04
208343_s_at	NR5A2	3.76	5.46	-3.24
210174_at	NR5A2	6.42	7.62	-2.29
208337_s_at	NR5A2	3.65	4.48	-1.77
208510_s_at	PPARG	3.73	5.47	-3.34
204021_s_at	PURA	5.35	5.98	-1.54
204020_at	PURA	8.13	8.61	-1.39
213806_at	PURA	3.4	3.79	-1.31
207785_s_at	RBPJ	5.49	6.53	-2.06
211974_x_at	RBPJ	7.78	8.59	-1.76
206443_at	RORB	3.38	3.62	-1.18
203135_at	TBP	4.07	5.17	-2.15
205287_s_at	TFAP2C	3.29	3.63	-1.27
205286_at	TFAP2C	3.33	3.61	-1.21

Continued

Transcript Cluster ID	Gene Symbol	Non-HCC Bi-weight Avg Signal (log2)	HBV-HCC Bi-weight Avg Signal (log2)	Fold Change (linear) (Non-HCC vs. HBV-HCC)
—	TFAP2D	—	—	—
205883_at	ZBTB16	7.31	6.08	2.35
—	ZNF354C	—	—	—

Note: — represent the expression of target gene was not found in the microarray of GSE14520.
